# Limited effectiveness of psychological inoculation against misinformation in a social media feed

**DOI:** 10.1093/pnasnexus/pgaf172

**Published:** 2025-05-28

**Authors:** Sze Yuh Nina Wang, Samantha C Phillips, Kathleen M Carley, Hause Lin, Gordon Pennycook

**Affiliations:** Department of Psychology, Cornell University, Ithaca, NY 14853, USA; Software and Societal Systems, Carnegie Mellon University, Pittsburgh, PA 15289, USA; Software and Societal Systems, Carnegie Mellon University, Pittsburgh, PA 15289, USA; Sloan School of Management, Massachusetts Institute of Technology, Cambridge, MA 02139, USA; Department of Psychology, Cornell University, Ithaca, NY 14853, USA

**Keywords:** misinformation, emotion, social media, psychological inoculation, psychology

## Abstract

Psychological inoculation is a promising and potentially scalable approach to counter misinformation. The goal of inoculation is to teach people to recognize manipulation techniques, such as emotional language, commonly found in misinformation online. While there is substantial evidence that inoculation increases technique recognition when directly assessed, it is not clear if this effect transfers to spontaneous detection of techniques and disengagement with the associated content in real-life contexts. In particular, emotional appeals are abundant on social media and known drivers of attention and engagement. Therefore, we examined the effects of emotional language and emotional manipulation inoculation on attention and engagement in a simulated social media feed environment. Through five preregistered studies, we found that inoculation only decreased engagement with emotionally presented content when we solely presented synthetic content relevant to the task of identifying emotional manipulation. Any addition of real tweets or even synthetic tweets containing other manipulation techniques (e.g. ad hominem attacks) into the feed appeared to nullify the effect. Our results highlight the importance of assessing misinformation interventions in ecologically valid contexts to estimate real-world effects.

**Significance Statement**

Combating online misinformation is an increasing necessity. One prominent intervention is psychological inoculation, which aims to teach people to recognize manipulative techniques commonly employed in online misinformation. Inoculation appears to be effective at improving people's ability to identify these manipulative techniques. However, it is unclear whether inoculation is effective at reducing engagement with misinformative content in real-life contexts. We test the effects of inoculating against one of these techniques, emotional language, in a simulated social media feed environment and find that inoculation has limited efficacy in these contexts.

## Introduction

Concerns about misinformation have led academics and technology companies to invest a great deal of resources in developing scalable interventions (1–6). Although several approaches have been proposed and tested (for reviews, see Refs. (3, 7, 8)), one increasingly popular approach is psychological inoculation (9–11). The logic of inoculation as an intervention against online misinformation is straightforward (12, 13): If users can be taught to identify the underlying markers of misinformation (e.g. the use of emotional language), they can better resist misinformation they later encounter online. Indeed, technology companies are already deploying inoculation interventions at scale (with millions of users receiving the intervention (14)), making inoculation perhaps one of the most widely adopted psychological interventions in recent history.

Despite the scale of the ongoing misinformation inoculation interventions, the state of the evidence supporting their efficacy in real-world settings is surprisingly limited (15). The vast majority of past research on inoculation in the context of misinformation has (justifiably) focused on validating the underlying logic of the intervention. In particular, researchers have developed different types of inoculations that focus on teaching participants to identify various elements (or “markers”) of misinformation. Unlike other prebunking interventions (i.e. interventions that aim to forewarn people of attempts to manipulate or deceive them), inoculation techniques aim to inform individuals about persuasion techniques that may apply to any number of misleading or false messages, rather than targeting any single narrative (16, 17). For example, gamified inoculations use engaging games to improve people's ability to identify misinformation techniques, such as evoking emotions and impersonation (18–20). Research focusing specifically on emotional manipulation has been shown to help people recognize that emotional language (e.g. content containing words like “horrifying” or “terrifying”) can be used to manipulate their attention (21).

However, one criticism of this research is that it tests psychological inoculation in a highly stylized environment: Participants are shown an intervention that lasts several minutes and then immediately given (often synthetic or researcher-created) stimuli that closely match the features that were the target of the intervention. For inoculation to be effective at combating misinformation at scale, it must be possible to deliver the intervention rapidly to many people. Furthermore, any beneficial effects should carry over to content that people actually encounter in their everyday lives.

**Fig. 1.**
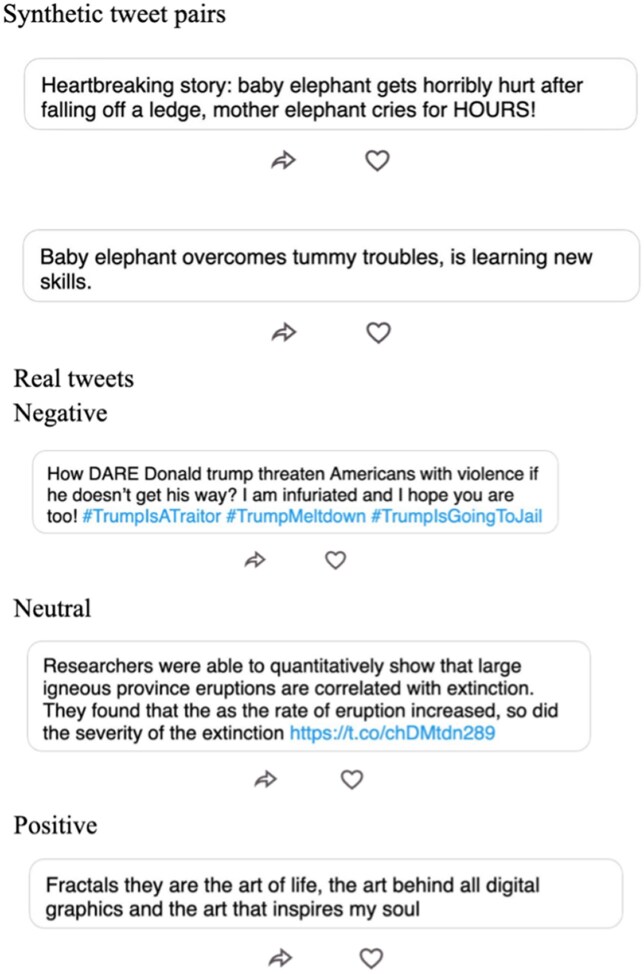
Examples of manipulative and neutral synthetic tweets (top row) and real tweets containing more than 50% negative, neutral or positive sentiment (bottom row).

To address these scalability concerns, a recent set of experiments by Roozenbeek et al. (22) tested a brief video version of the intervention. The researchers tested five different inoculation interventions that targeted different types of misinformation techniques: emotionally manipulative language, incoherence, false dichotomies, scapegoating, and ad hominem attacks. To demonstrate feasibility, they found that presenting participants with the video improved their ability to distinguish between synthetic social media posts that contained the targeted manipulation technique or were neutral (see Fig. 1). To evaluate real-world efficacy, they also conducted a quasifield experiment where YouTube users watched one of two inoculation videos (emotional language and false dichotomies) in the course of their everyday web browsing. However, as with previous studies, the efficacy of the intervention was tested by directly asking users (via a measurement method used in market research) to identify whether the targeted misinformation technique was present in a synthetic social media post. This is true of other large-scale tests of scalable inoculation that have been reported, such as a campaign by Google that reached 21 million Germans (23). Although the interventions were delivered in the field (i.e. on YouTube), the outcomes were measured in the same way as a typical online experiment where users are asked directly about the manipulation technique. Thus, while it appears that these brief video interventions increase participants' ability to identify manipulative content when asked to evaluate synthetic claims, it is unclear if users who have been inoculated are able to *spontaneously* apply what they have learned to the real-world misinformative content.

Indeed, there are reasons to believe that the ability to identify manipulative techniques in controlled experiments may not translate to benefits in real-life contexts (24, 25), especially when the experiments used synthetic stimuli (22) (i.e. stylized social media posts created by researchers to specifically manipulate the presence/absence of emotional language). The transfer problem, a long-standing issue in research (26–29), highlights the difficulties in applying learned knowledge or skills to new contexts (30). That is, for inoculation to be effective and useful in real life, people have to not only learn and remember the content in the inoculation intervention, but also be able to spontaneously apply that learned knowledge to identify manipulative content in their everyday lives (e.g. when browsing the internet or social media). This may be a uniquely salient problem on social media, as it is a highly distracting context with a vast amount of content competing for people's attention (31). In fact, recent research indicates that the social media context may specifically hinder critical thinking (32).

We therefore examined whether a brief emotional manipulation inoculation video intervention (i.e. the same intervention in (22)) reduces engagement with emotional content in a more realistic setting—a simulated social media feed environment. We focus on emotional language because a large body of work suggests it influences attention and engagement on social media (33–36), and no work has systematically evaluated the effects of inoculation on attention and engagement with emotional content in a context where the intervention needs to be spontaneously applied. We also compared the effects of inoculation on real-world tweets curated for this work (37) and synthetic stimuli from Ref. (22) to investigate the effectiveness of inoculation on more ecologically valid content.

### The present research

We conducted five preregistered studies (see Table 1) to examine the effects of emotional language and a brief video inoculation against emotionally manipulative language on Yourfeed, a research social media platform that presents posts in a scrollable feed (38). This platform allowed us to reproduce the real-world attention dynamics users experience when they browse social media feeds (39), and it provided metrics of attention and engagement that are exploited by algorithms. For example, ranking algorithms often amplify emotionally charged content (40), which captures attention and drives engagement (e.g. retweets) (33). We therefore measured three outcome variables on the platform: dwell time, likes, and shares. We treat dwell time on each post as a proxy for attention, and treat liking and sharing as a measure of engagement. Importantly, although this simulated social media environment is much closer to “real-world” social media than prior research on inoculations, it nonetheless represents a fairly liberal test of the hypothesis: If inoculation is ineffective in the context of a research study using a simulated feed, it is quite unlikely to be effective in an actual feed and during the course of an individual's everyday life where attention is weaker and distractions are presumably even greater.

**Table 1.** Study details.

	*N*	Stimuli	No. of stimuli in feed	Video	Preregistration
Study 1	974	Real-world	120	No	https://aspredicted.org/rhpg-pzbv.pdf
Study 2	953	Real-world	120 (40 pre/80 post)	Yes	https://aspredicted.org/qyfv-x9sn.pdf
Study 3	962	Real-world	80 (20 pre/60 post)	Yes	https://aspredicted.org/cwj3-gbjk.pdf
Study 4	951	Synthetic	80 (20 pre/60 post)	Yes	https://aspredicted.org/vzr3-ch47.pdf
Study 5	966	Synthetic	54 (27 pre/27 post)	Yes	https://aspredicted.org/xyr6-3xty.pdf

Real-world stimuli are from Ref. (37) and synthetic stimuli are from Ref. (22).

Study 1 (*n* = 974; recruited from Prolific) investigated whether emotional language in real-world English tweets (37) affects attention and engagement in a simulated social media feed environment. The goal of this study was to establish that the dynamics found in real-world social media contexts (where emotional content garners greater engagement) are replicated in our simulated news feed. Studies 2 to 5 (total *n* = 3,832; recruited from Prolific) were randomized controlled experiments that examined the effects of a brief, scalable inoculation video (https://inoculation.science/inoculation-videos/emotional-language/) that teaches people about emotional manipulation, a technique that is thought to be commonly found in misinformation (13, 16). This video has been used in previous inoculation experiments and has also been deployed at scale (14, 22). We focus on video inoculation (as opposed to something like the gamified inoculations used in Refs. (9, 41)) because, unlike gamified inoculation treatments, these treatments are specifically designed to be scalable and can therefore be easily integrated into social media feeds.

Although these interventions can target various manipulative tactics (e.g. ad hominem, scapegoating), we focus on emotional language because inoculation had some of the largest and most consistent effects on identifying emotional language (22). Additionally, we used techniques from natural language processing to measure the amount of emotional language in real social media posts, allowing us to test the effects of inoculation beyond synthetic, researcher-generated stimuli. Emotional content is also likely to be highly prevalent on social media, particularly given past work suggesting emotional content garners greater engagement and diffusion (33, 42, 43). Note that, as in Ref. (22), we investigate the impact of inoculation on engagement with emotionally evocative content in particular without making any additional claims about whether the content contains misinformation per se.

To vary the realism and ecological validity across the four experiments, we populated the feed in each experiment with either real-world tweets (studies 1 to 3) (37) or synthetic stimuli (studies 4 and 5) used in prior inoculation experiments (22). Figure 1 features examples of real and synthetic tweets as they are presented to users on Yourfeed. Real-world tweets have greater ecological validity, but emotionality in these tweets may not necessarily reflect emotional *manipulation* per se. Synthetic stimuli, on the other hand, lack realism but were designed by experts to contain clear examples of emotional manipulation (and have been used in prior experiments that successfully shown inoculation can increase manipulative technique recognition (22)).

In all five experiments, participants were instructed to engage with posts as they would on a real social media platform. In studies 2–5, participants were randomly assigned to watch either the inoculation video or one of three control videos of approximately equivalent length (about 1.5 min). After scrolling through less than half of the simulated feed (see Table 1 for details), participants are shown the video treatment. To ensure participants watched the video, the video could not be skipped and participants could not continue with the study until it finished playing. After watching the video, they continued scrolling until they reached the end of the feed.

To assess whether the inoculation treatment led to decreased engagement (likes, shares) and attention (dwell time) with real content high in emotional language (studies 2 and 3) and synthetic content designed to contain emotional manipulation (studies 4 and 5), we ran three models per study For studies 2 and 3, we ran ordinary least squares regressions predicting (log) dwell time, logistic regressions predicting like or not, and logistic regressions predicting share or not as a function of the interaction between inoculation treatment and percent emotional language with clustered standard errors for participants and posts. For studies 4 and 5, we ran the same models as a function of the interaction between inoculation treatment and the presence of emotional manipulation. That is, these interactions between condition (treatment/control) and manipulativeness represent discernment of manipulative content.

## Results

To assess the external validity of the studies on the synthetic social media feed (hereafter “Yourfeed”) and the realism of the two different stimulus sets, we begin by comparing engagement rates. For example, recent field studies found that Facebook users clicked on 8.3% of the content they were exposed to (44). Participants in studies 1 to 3 (which used real-world tweets from Ref. (37)) clicked on 13.3% of the content they saw, whereas participants in studies 4 and 5 (which used synthetic tweets from prior work (22)) clicked on 19.9% of the content they saw (i.e. more than two times the click rates in real-world field data).

We assess whether emotional content garners greater engagement and attention on Yourfeed. To measure emotional language, we utilize a RoBERTa model fine-tuned on sentiment analysis on tweets (45), which outputs the percentage of negative, neutral, and positive sentiment in each tweet. We find that for real-world posts (studies 1 to 3), negative emotional language is associated with longer dwell times and more shares, whereas positive emotional language is associated with more likes (BF_10_ > 1.5 for all; see SI and Fig. S1). These results suggest that emotional language affects engagement on Yourfeed as it does on real social media platforms (e.g. 42, 33, 43) (see also (46)) which suggests negative language is associated with greater shares and positive language is associated with greater likes. We do not, however, find clear associations between emotional language and attention or engagement in synthetic tweets (studies 4 and 5; see SI).

### Inoculating against emotional language manipulation

**Fig. 2.**
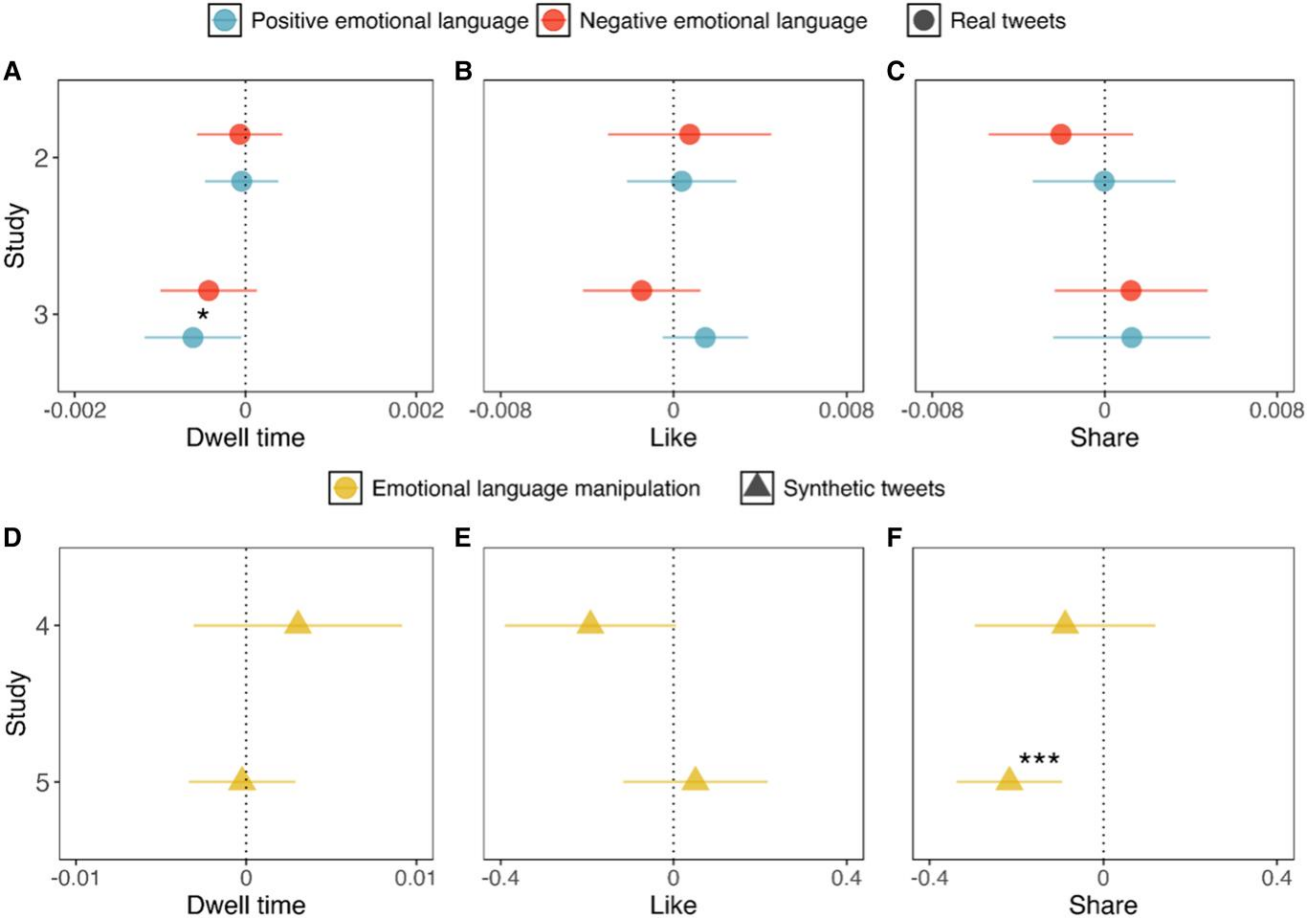
Regression coefficients for the interaction between inoculation and positive or negative emotional language for studies 2 and 3 (top row) in models predicting dwell time (A), likes (B), and shares (C). Regression coefficients for the interaction between inoculation and emotional manipulation for studies 4 and 5 which used synthetic stimuli (bottom row) in models predicting dwell time (D), likes (E), and shares (F). Error bars indicate 95% CI. *P < 0.05, **P < 0.01, ***P < 0.001.

Does the inoculation video decrease engagement with and/or attention toward emotional content? Figure 2 shows the interaction effects between the inoculation treatment and the positive and negative sentiment scores for studies 2 and 3. Overall there was very little evidence that inoculation had a meaningful impact. In particular, we found inoculation decreased dwell time on posts that contain more positive emotional language (*b* = −0.00062, BF_10_ = 0.09, *P* = 0.034) in study 3, although this effect disappears when the analysis was restricted to the most attentive participants on Yourfeed (i.e. those who did not switch browser tabs during the inoculation treatment; see robustness checks in SI). The effect of inoculation on dwell time on posts that contain more negative emotional language in study 3 was not significant (*b* = −0.00043, BF_10_ = 0.026, *P* = 0.13). The remaining interactions between inoculation and emotional language across outcomes are null (BF_10_ < 0.03 for all), suggesting that the inoculation treatment essentially did not affect attention toward or engagement with emotional tweets. Studies 2 and 3 differed only in the length of the feed (120 posts in study 2; 80 posts in study 3); this had no apparent impact on the (lack of) treatment effect.

Since studies 4 and 5 used synthetic stimuli from prior work (22), we use researcher-generated labels of which messages contained the emotional language manipulation and which were neutral. We include analyses using the amount of emotional language measured in the post by sentiment analysis in the SI (see Fig. S2). In study 4, we populated the feed with tweets that used different manipulation techniques (i.e. emotional language, ad hominem attacks, false dichotomies, scapegoating, and incoherence)—although we focus on the results for the emotional language tweets. In study 5, the feed was further simplified to only contain the synthetic tweets relating to emotional language manipulation. Importantly, we find that the inoculation treatment reduced engagement (i.e. sharing) only in study 5 (*b*  *=* −0.22, BF_10_ = 6.05, *P* < 0.001, Cohen's *d* = 0.16; Fig. 2F), but not in study 4 (BF_10_ = 0.02, *P*  *>* 0.1). In study 4, the inoculation treatment marginally decreased likes on posts that contain emotional language manipulation (*b*  *=* −0.19, BF_10_ = 0.09, *P* = 0.057, Cohen's *d* = 0.12), although this effect did not hold when we restricted the analysis to the most attentive participants or participants who correctly responded to attention checks (*P* = 0.3; *P* = 0.096, respectively). Inoculation did not influence likes in study 5 (BF_10_ = 0.01, *P* > 0.55). These results suggest that the inoculation treatment was only effective in reducing engagement (i.e. sharing) with manipulative tweets when the feed contained only emotionally manipulative posts. In particular, when the feed contained other types of manipulative content (other than emotional language), the inoculation treatment failed to reduce the sharing of emotionally manipulative posts.

There are no significant effects of inoculation on dwell time or likes for study 4 or 5 (BF_10_ < 0.1 for all), and this was true even in the case where inoculation led to a reduction in engagement (i.e. shares in study 5). However, when we restricted the analysis to the most attentive participants in studies 4 and 5, inoculation decreased dwell time on emotionally manipulative posts in study 5 (*b* = −0.0096, *P* = 0.049; see SI for full models). In contrast, though, when we remove participants that incorrectly responded to attention checks, inoculation *increased* dwell time on emotionally manipulative posts in study 4 (*b* = 0.0086*, P* = 0.007) and study 5 (*b* = 0.0029, *P* = 0.043). The results on dwell time were, therefore, inconclusive.

We found no significant effects on likes even when restricting analyses to the most attentive participants. For study 4, we also ran the same models for the other four types of manipulation represented in the stimuli, where we find some (weak) evidence that inoculation also affects engagement with posts containing other manipulative techniques (see SI for full models).

Taken together, studies 2 to 5 demonstrate that inoculation against emotional manipulation only decreased engagement on posts containing emotional manipulation in particularly artificial and restricted contexts: when the feed contained synthetic stimuli from prior work *and* included *only* emotionally manipulative content (study 5). But when the feed contained synthetic stimuli with other manipulative techniques (study 4) or real-world tweets (studies 2 and 3), inoculation was no longer effective in reducing engagement with emotionally manipulative content. Except where otherwise noted, these results are robust to removing people who failed attention checks or were inattentive during the inoculation treatment (see Robustness checks in the SI). Furthermore, posts containing emotional language and the emotional language manipulation technique have differential effects on attention and engagement, suggesting a disconnect between the two constructs (see SI for analysis of the effect of inoculation on emotional language in synthetic posts).

## Discussion

Across multiple studies with 3,881 participants, we find that inoculation against emotional language manipulation is not effective in an ecologically valid context. That is, the beneficial effects of inoculation reported in prior work might not generalize beyond controlled laboratory experimental settings to more realistic contexts where people are exposed to a more diverse range of content and in social media environments where attention is more fickle. Importantly, whereas past work has focused on whether the inoculation improves manipulative technique recognition, we focused on more ecologically valid outcomes—whether it affects attention to and engagement with emotional content—and showed that it is ineffective when deployed and evaluated in more realistic contexts.

By presenting posts in a scrollable feed that mimics people's actual social media experience, we further increased the realism of our experiments and avoided directing participants' attention to certain posts or features thereof, which has been shown to influence observed behavior (47). When the emotional inoculation treatment was delivered on our mock social media feed, it did not reduce engagement with real-world tweets containing emotional language (studies 2 and 3) or with synthetic emotionally manipulative posts when mixed with synthetic posts containing other kinds of manipulative techniques (study 4).

The only case where inoculation was effective at reducing sharing of emotional content was study 5, which was designed to match as closely as possible a typical controlled experiment where participants are exposed only to either neutral or emotionally manipulative synthetic content (22). The main difference to prior inoculation studies is that content was presented in the context of a social media feed instead of as individual posts that were presented one at a time. Although it was not their focal outcome, Roozenbeek et al. (10) did report an effect size of *d* = 0.21 for the effect of emotional language on sharing in particular. Interestingly, this is fairly similar to our effect size in study 5, with a Cohen's *d* of 0.16. Thus, it may be that the addition of the distracting *content* in our simulated social media feed played a particularly important role in undermining the inoculation effect (as opposed to simply putting the content in the form of a scrollable social media feed).

Inoculation did not significantly affect whether participants “liked” posts containing emotional language (manipulation) across studies 2 to 5 (BF < 0.1 for all). Furthermore, it failed to conclusively influence dwell time even when shares were decreased on emotionally manipulative posts (i.e. study 5). There was some evidence in study 3 that inoculation decreased dwell time on positive language (*P* < 0.001, BF = 0.09), but this was not present in any other studies and the effect disappeared in robustness checks. We expected that inoculation, if effective, might either *decrease* people's attention to emotional content by teaching them that emotionality is a sign of potential manipulation that should not be paid attention to, or *increase* people's attention by making emotionality salient. We find that inoculation consistently did not influence attention towards real-world posts containing more negative emotional language or towards synthetic emotionally manipulative posts. This suggests people are not automatically disregarding emotional posts following inoculation, nor are they paying special attention towards them.

One possible interpretation of our null effects is that inoculation only works on very clear examples of the technique in question—however, this interpretation is at odds with our results in study 4, where we had clear examples of emotionally manipulative tweets but did not find an inoculation effect. Relating to this, there is evidence that inoculation interventions are less effective without some form of evaluation (“posttest”) immediately afterward (48, 49). The feed following the inoculation treatment in study 5 most closely mirrors typical posttests in which participants solely differentiate between messages with and without emotional language manipulation. Although participants are not explicitly evaluated in these cases, it might be that the inclusion of clear examples (without distractors) helps participants consolidate what they have learned from the inoculation. In other words, the inoculation effect—at least in this particular form—may be undermined by the presence of content that is not directly related to the target of the inoculation. Given that users have extremely idiosyncratic news feeds, this suggests that the bar for an inoculation to have a real-world impact may be very high.

Some past work has evaluated the impact of inoculation in ecologically valid contexts and found different results; however, there are important differences in methodology that require some emphasis. As mentioned earlier, large sample field experiments where users were shown inoculation videos (including the inoculation we used here) on YouTube were tested using “pop-up” questions that did not require participants to spontaneously apply the inoculation (but instead prompted them with a direct question about manipulativeness (22, 23)). Another example is research where a simulated social media platform was used, but the manipulative content was nonetheless presented on a single static screen (albeit with social-media-style engagement buttons) (50). This, too, does not share the critical component of YourFeed, where users must spontaneously apply the inoculation; i.e. since there is only one piece of content on the screen at a time, it is clear to the participant that they should be applying what they learned in the inoculation to the specific piece of content that they are being shown. Once this is made less obvious, as in our study 4 where the target of the inoculation was intermixed with other content, it may become much more difficult for users to spontaneously apply the inoculation.

Our results are consistent with past work highlighting the difficulty of transfer (26–29). That is, it seems difficult for participants to transfer what they have learned in the inoculation video to contexts where they must spontaneously apply this knowledge. It is also possible that more tightly controlled contexts (such as in study 5) create demand effects, whereby participants may understand that the intention is for them to engage less with emotionally manipulative content. However, we would argue that this intention is clear even in our other studies: the inoculation video contains specific messaging about not being manipulated, for example. Rather, the difficultly arises in applying what they have learned from the inoculation in contexts where cases of emotional manipulation are less obvious.

We view this work as a more ecologically valid test of inoculation theory, but our studies are not without limitations. We put measures in place to ensure participants paid attention to the inoculation video presented (e.g. preventing participants from closing the video window until the video has finished playing), but it is not possible to verify that participants fully attended to the treatment video. Despite this concern, inattention to the intervention is a real-world limitation as well. Indeed, we tested the inoculation video in a context even more favorable than it would be presented on an actual social media feed. In studies 3 to 5, we presented the video treatments as unmuted by default—which is not the norm for video content on most social media sites—to increase the likelihood that participants would attend to the treatment. We also conducted robustness checks and removed participants who switched tabs during the video as a proxy for attentiveness to the inoculation treatment (although note that participants who did not change tabs may not have attended to the video, and some who changed tabs may have continued listening to the video). None of these additional checks were sufficient to yield positive results of the inoculation treatment. It is also possible that the longer inoculation treatment would have had stronger effects, however, given that attention is already an issue even with the shorter video treatment deployed here, we predict that holding participant's attention for this longer treatment may be even more challenging and less scalable to real-world contexts. In addition, we did not incorporate a delay between the inoculation intervention and outcomes, which also likely decreases the effectiveness of inoculation further.

Another limitation is that there are also likely differences in how people interact with our mock social media platform in comparison to real platforms. However, past work suggests that, for example, intentions to share are correlated with real sharing behavior (51). Furthermore, it is not obvious why this would undermine the inoculation intervention in particular. And, regardless, we did replicate the standard effect when the simulated feed was populated entirely with synthetic posts that were specific to the inoculation intervention (as in past work). Similarly, as we focus on outcomes that have real-world impact (i.e. whether inoculation reduces engagement with emotional content) and avoid drawing participant's attention to evaluating specific claims for greater ecological validity, we do not test whether we replicate past work suggesting inoculation improves people's ability to identify manipulative techniques (e.g. 22) or explicitly ask participants about their belief in manipulative claims. However, the finding that inoculation reduces sharing of emotionally manipulative content in study 5 (which most closely matches past work) suggests that inoculation was effective in this specific context.

Overall, we present evidence that inoculation does not reduce engagement with emotional content and that the benefits of inoculation found in controlled experiments do not transfer to more realistic contexts. Given that these interventions have already been deployed in the field by technology companies (14), our results highlight the need to rigorously reevaluate the efficacy of existing inoculation interventions in ecologically valid contexts before deploying them at scale in the field.

## Materials and methods

We conducted five preregistered studies on Yourfeed (38), a mock social media platform. All studies were approved by the Cornell University Institutional Review Board for Human Participants (IRB0147753). All preregistrations are available on OSF: https://osf.io/3k7d6/. Informed consent was obtained from all participants. All studies were conducted via CloudResearch Connect (see Table 1; attrition details is in Table S1 and information on sample demographics is in Table S2). In all studies, participants completed two sections: a Qualtrics survey with demographic questions and individual difference measures plus the social media sharing section on Yourfeed. When they landed on Yourfeed, they saw the following instructions: “Thank you for participating! Next, you will see a social media newsfeed with [120/80/54] posts. Please browse this newsfeed like you usually would for social media. For each post, indicate whether you would share it with your network and like/favorite it.”

In study 1, participants first completed the Yourfeed section and then the Qualtrics survey. In this study, we examined the effects of emotionality on real-world tweets, drawn from Ref. (37). In studies 2 to 5, participants first completed the Qualtrics survey and then the Yourfeed section. In these four studies, we tested the efficacy of a brief (under 2 min in duration), animated inoculation video on emotional language from Ref. (22), with variations in the feed content and length in each study. Participants were randomly assigned to either the treatment (inoculation) group that watched the emotional language inoculation video partway through the social media feed, or a control group that watched a video of similar length and esthetic, but with content unrelated to online misinformation or emotionality (specifically, the control videos were either about bananas, freezer burn, or curling). Participants continued scrolling through the feed and interacting with posts after the video and could not proceed until the video played through.

In study 2, to mimic real social media environments, the video was muted upon launch and participants were asked to unmute the video before playing. In studies 3 to 5, to increase likelihood that participants would attend to the video, the video was unmuted by default. Additionally, we decreased the length of the feed from 120 posts in studies 1 and 2 to 80 posts in studies 3 and 4 to increase attention toward the entire feed (Table 1). Finally, in study 5, we sought to eliminate potential distractions and interference from the presence of other manipulative techniques: We showed participants mostly posts from the emotional manipulation category (both manipulative and neutral posts) after the video, and only showed nonmanipulative stimuli before the video.

### Stimuli

In studies 1 to 3, we pseudorandomly selected 20,000 stimuli from a large database of tweets that were posted within a 24-h time period starting on 2022 September 21 (37). From these, we selected English-only tweets to get a sample of 6,317. We then applied sentiment analysis using roBERTa fine-tuned on tweets (45). The model returns the percent of the given text that contains positive emotion, negative emotion, or is emotionally neutral (i.e. sentiment) (52). We then selected 240 posts in total: 60 tweets that contained more than 50% positive sentiment, 60 tweets that contained more than 50% negative sentiment, and 120 tweets that contained more than 50% neutral language. We used the following criteria: (i) no inappropriate content (e.g. sexual content or profanity), (ii) not deleted or removed from X, to allow us to compare actual engagement with engagement on Yourfeed, and (iii) the tweet contained enough context to make sense in isolation. For each participant in studies 1 to 3, we randomly selected tweets from these 240 tweets to curate their feed. The topic of the tweets varies widely, including United States and international politics, cryptocurrency, life advice, travel, and religion.

For studies 4 and 5, we used stimuli from (22), making minor adaptations to fill in information (e.g. names) that was blacked out in the original stimuli so that posts would look natural in a social media feed. In study 4, we included manipulative and nonmanipulative posts from all five techniques in Ref. (22), with emotional language technique items appearing only after the video. In study 5, all emotional language technique items also appear after the video and all other posts in the feed are nonmanipulative. In other words, the only manipulative content included in study 5 contain the emotional manipulation technique. In all studies, the order in which stimuli was presented was randomized.

### Outcome measures

Yourfeed collects dwell time information based on how long a post is fully displayed on a participant's display (38). There are a few sections of the feed where dwell times may be inflated: (i) When participants first landed on the feed, they had to read the initial instructions and acclimatize to the feed, thus dwell time for the first few posts will be longer; (ii) when the video modal/pop-up was shown on the display, the posts in the background were considered as being “dwelled on,” and therefore have inflated dwell times; and (iii) dwell time for posts at the very end of the feed will also be inflated as participants slowed down to click the button to submit and proceed to the next section of the study. To account for these issues (see Ref. (38)), we removed these posts from all analyses: those at the beginning and end of the feed and those that were on screen (in the background) while the video popup was on screen. Because participants could scroll back up and view posts more than once, for each participant, we summed the dwell times for each post.

We also analyzed likes and shares (shown as two separate buttons on Yourfeed) as two distinct binary variables indicating engagement—whether the participant clicked on the like/share button for that post, regardless of whether they later clicked the button again and “undid” the like/share. That is, we consider any click on the like/share button as a form of engagement (see Table 2 for mean engagement statistics).

**Table 2.** Average dwell time, number of likes, and number of shares per post or participant for each study, with 95% winsorization of dwell times and removal of buffer posts.

Study	Dwell time (s) per participant/post	Likes per post	Shares per post	Likes per participant	Shares per participant	Posts analyzed per participant
1	6.34 (7.58)	46.45 (26.83)	26.43 (14.09)	11.46 (16.91)	6.52 (13.17)	110
	3.18 (3.04)	38 (14.0)	23 (8.0)	6 (6.0)	1 (1.0)	
2	4.3 (5.9)	17.33 (12.81)	10.33 (6.85)	5.15 (7.67)	2.6 (6.93)	70
	1.62 (1.59)	13 (6.0)	9 (4.0)	3 (1.0)	0 (0.0)	
3	6.65 (7.57)	20.58 (14.1)	11.32 (7.69)	5.13 (7.67)	2.82 (6.08)	51
	3.7 (3.38)	16 (7.0)	9 (4.0)	3 (3.0)	0 (0.0)	
4	16.83 (14.54)	63.3 (32.4)	38.27 (29.99)	6.73 (10.07)	4.067 (7.6)	49
	14.02 (10.29)	53 (17.0)	26 (7.0)	3 (3.0)	1 (1.0)	
5	17.35 (14.27)	133.55 (47.33)	119.05 (35.44)	2.7 (4.08)	2.41 (4.04)	20
	14.59 (9.37)	112.5 (23.0)	109 (14.5)	1 (1.0)	0 (0.0)	

For each study, means and SD are shown in the first row, and medians and median absolute deviations are shown in the second row. Note: No treatment or videos were shown in study 1.

### Analyses

We fitted regression models in all studies. The regressions predicting (log) dwell time were ordinary least squares models, while the regressions predicting engagement (share or not, like or not) or not were logistic models.

For study 1, we modeled each outcome as a function of the percent of positive and negative emotional language contained in each post, with standard errors clustered on participants and posts. For studies 2 to 5, we modeled each outcome as a function of the treatment condition, emotional language (i.e. percent of positive and negative emotional language in each post), and the interaction between inoculation treatment and emotional language, with clustered standard errors for participants and posts. Full model outputs are reported in Tables S3–S9.

For studies 4 and 5 (which used only synthetic stimuli), we modeled each outcome as a function of the treatment condition, emotional language manipulation label (from Ref. (22)), and the interaction between treatment and emotional manipulation, with standard errors clustered on participants and posts. In study 4, which included posts containing other manipulative techniques for which inoculation is tested in Ref. (22) (ad hominem attacks, false dichotomies, scapegoating, and incoherence), we also tested whether engagement with these other types of manipulation was affected by the emotional language inoculation video (see Tables S23–S26 and S35–S41).

For studies 2 to 5, we ran two preregistered robustness checks for each model. First, we refitted the models above after excluding participants who responded incorrectly to an attention check on Qualtrics (see Tables S10–S15). Second, we refitted the models above after excluding participants who switched tabs during the treatment or control video (see Tables S16 and S18–S22). For study 2, we also refitted the models above after excluding participants who did not unmute the video during its duration (see Table S17). Unfortunately, we deviated from study 1 preregistration as due to experimenter error attention checks were not included in the survey. Therefore, we cannot do robustness checks by running the models with participants who failed the attention check removed.

We calculated adjusted fractional Bayes Factors with Gaussian approximations for the primary models (Tables S3–S9 in SI) using the BFPack R package (53). We report BF_10_ for each estimate where the alternative hypothesis is directional based on the sign of the estimate (i.e. *b* < 0, *b* > 0) and the null hypothesis is *b* = 0. Thus, if BF > 1, the evidence is more consistent with the alternative hypothesis; if BF < 1, the evidence is more consistent with the null hypothesis.

To compare effect sizes with those reported by Ref. (22), we ran an ANOVA using the R package “afex” (54) for the effect of inoculation on sharing for study 5, controlling for the mean preintervention shares for each participant. This allowed us to calculate a Cohen's *f* value which we then converted to Cohen's *d* to match (22).

We report detailed attrition information in the SI for studies 1 to 5 (see Supplementary Text and Tables S27–S34). We do not find evidence of different attrition across the treatment and control groups in studies 2 to 5 (χ^2^ < 0.43, dof = 1, *P* > 0.51 for all). Nevertheless, we checked whether attrition differed on any pretreatment covariates. With one exception (longer average pretreatment dwell time per post is associated with lower likelihood to attrit in study 4), we do not find evidence of attrition bias.

## Supplementary Material

pgaf172_Supplementary_Data

## Data Availability

All data and code needed to replicate this analysis are available at: https://osf.io/3k7d6/ (55).

## Preprints

This manuscript was posted as a preprint: https://osf.io/preprints/psyarxiv/uxp85_v1.
