# Supplementary material for: Limited effectiveness of psychological inoculation against misinformation in a social media feed
Source: PNAS Nexus. 2025 May 28;4(6):pgaf172. doi: 10.1093/pnasnexus/pgaf172 (PMC12134459; doi:10.1093/pnasnexus/pgaf172)
Supplement: pgaf172_Supplementary_Data [file pgaf172_supplementary_data.pdf]

Supplementary Materials for

Limited effectiveness of psychological inoculation against misinformation in a social media feed

Sze Yuh Nina Wang *et al.*
*Corresponding author. Email: szeyuhwang@gmail.com

Includes:

Supplementary Text
Survey Measures
Figs. S1 to S2
Tables S1 to S39

Supplementary Text

Attrition

We report detailed attrition information in Table S1 for Studies 1 to 5. In most cases, we do not know if psychological or technical factors drove the attrition. We do not find evidence of different attrition across the treatment and control groups in Studies 2 to 5 ($\chi^2 < 0.43$, df = 1, $p > 0.51$ for all). Nevertheless, we checked whether attrition differed on any pre-treatment covariates (see Tables S27-S34 for model outputs).

We ran a binomial logistic regression predicting study completion as a function of all pre-treatment variables except ethnicity. Because there are 92 comparisons across logistic regressions for Studies 2 to 5, we applied a Bonferroni correction and designated p-values less than 0.05/92 = 0.00054 as significant. We found longer average dwell time on posts before the treatment video was associated with completing Study 4 (p = 0.00009). Longer dwell time on average can be interpreted as expending more cognitive effort into evaluating posts and therefore, more effort given to the study overall. Therefore, it is relatively unsurprising that longer dwell time is associated with less attrition (in at least one study). With this slight exception, we do not find evidence of attrition bias.

Table S1. The number of participants who left at each stage of each study and completed each study.

	Study 1	Study 2	Study 3	Study 4	Study 5
Approved in Cloud Connect + no participation	33	1	6	50	3
Qualtrics only		3	2	0	0
Yourfeed + no Qualtrics	0				
Qualtrics + quit Yourfeed before video		5	1	0	0
Qualtrics + no video + completed Yourfeed		1	1	3	14
Qualtrics + quit Yourfeed after video		30	19	10	9
Other technical error in Yourfeed	2	5	4	13	5
Completed study	975	953	962	941	988

Comparison of the effect of emotional language on the dwell time, liking, and sharing of real-world and synthetic tweets

We observe a different pattern of associations between emotional language and dwell time, liking, and sharing for real-word (Studies 1-3) and synthetic posts (Studies 4 and 5): We find that for real world posts, negative emotional language is associated with longer dwell times and more shares, whereas positive emotional language is associated with more likes. Conversely, synthetic posts with emotional language (regardless of positive or negative) had shorter dwell times (Fig. S1A), but did not consistently affect engagement, with the exception that positive emotional language is associated with lower likelihood to share posts in Study 5 (Fig. S1C). These results are robust to removing inattentive participants with the exception that negative emotional language is no longer associated with less dwell time in Study 4 and positive emotional language is no longer associated with lower likelihood to share in Study 5.

Taken together, we find emotional language affects dwell time and engagement differently in synthetic and real-world tweets. Broadly, emotional language increases attention to and engagement with real-world tweets (Studies 1 to 3), but it has no clear effects on attention and engagement with synthetic tweets (Studies 4 and 5).

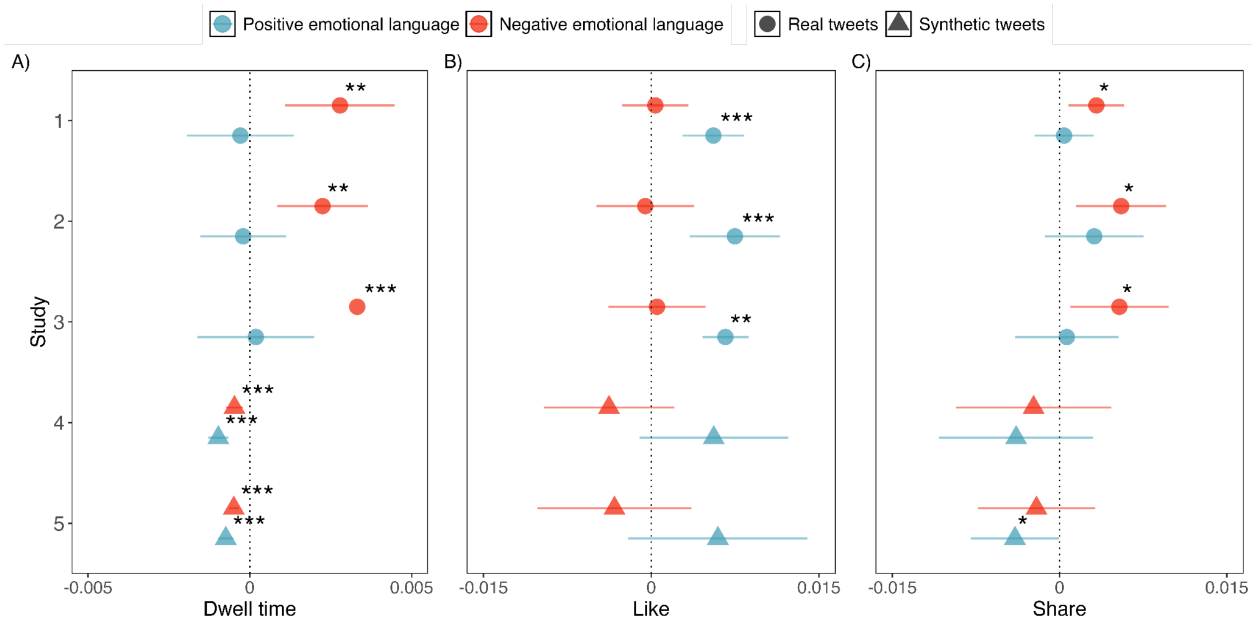

Fig. S1. Regression coefficients for the effects of positive and negative emotional language. Positive and negative emotional language were quantified using sentiment scores from a fine-tuned roBERTA model (Barbieri et al., 2020). For each study, we ran an OLS regression predicting (log) dwell time (A) and logistic regressions predicting like or not (B) and share or not

(C), as a function of emotional language or (when applicable—in studies 2 to 5) the interaction between inoculation treatment and emotional language, with clustered standard errors for participants and posts. Error bars indicate 95% confidence intervals. Studies 1 to 3 used real-world tweets whereas studies 4 and 5 used synthetic stimuli from prior work (Roozenbeek et al., 2020). *p<0.05, **p<0.01, ***p<0.001.

<u>Interactions between inoculation and emotional language in synthetic posts</u>

Figure S2 contains regression coefficients for the interaction between inoculation and positive or negative emotional language for Studies 4 and 5. In Study 4, where the feed contained synthetic stimuli with various manipulative techniques, inoculation successfully decreased likes on posts that contain more positive emotional language (b = -0.0042, p = 0.0065). Surprisingly, this effect is null when restricted to the most attentive participants. In Study 5, where the feed included only emotionally manipulative or neutral posts, inoculation increased dwell time on posts that contain more positive (b = 0.001, p = 0.014) or negative emotional language (b = 0.001, p = 0.003). It does not, however, affect shares or likes on posts containing more emotional language.

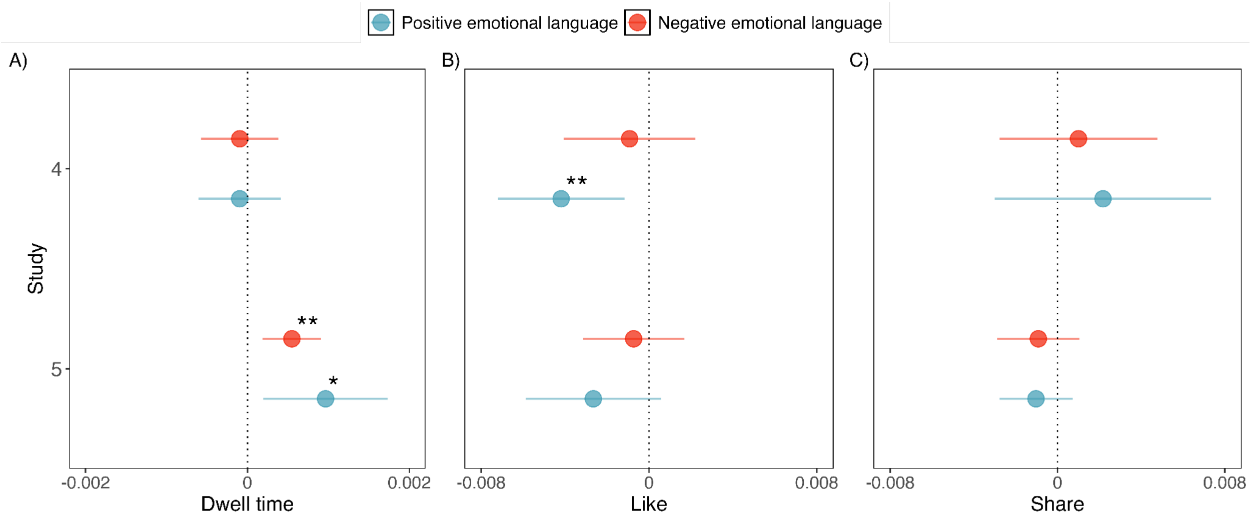

Figure S2. Regression coefficients for the interaction between inoculation and positive or negative emotional language for studies 4 and 5. Positive and negative emotional language were quantified using sentiment scores from a fine-tuned roBERTA model (Barbieri et al., 2020). For each study, we ran an OLS regression predicting (log) dwell time (A) and logistic regressions predicting like or not (B) and share or not (C), as a function of the interaction between inoculation treatment and emotional language, with clustered standard errors for participants and posts. Error bars indicate 95% confidence intervals. Studies 4 and 5 used synthetic stimuli from prior work (Roozenbeek et al., 2022)*p<0.05, **p<0.01, ***p<0.001.

Survey Measures

All participants answered a standard battery of demographic questions, as follows:

- What is your age?
- Which of the following best describes your gender identity/identities? (Select all that apply.) (Male/female/non-binary/prefer to self-describe)
- Please indicate the answer that includes your entire household income in 2023 before taxes. (Brackets of $10,000 increments)
- What is the highest level of school you have completed or the highest degree you have received? (Less than high school degree/high school graduate (high school diploma or equivalent including GED)/some college but no degree/associate degree in college (2-year)/Bachelor's degree in college (4-year)/Master's degree/Doctoral degree/Professional degree (JD, MD)
- Please choose whichever ethnicity you identify with (you may choose more than one option). (White/Caucasian, Black or African American, American Indian or Alaska Native, Asian, Native Hawaiian or Pacific Islander, Other)
- How much would you say you believe in God or Gods? (7 point Likert from Not at all/Very much)
- Economic conditions in the USA today are … (7 point Likert from Poor to Excellent)
- Which of the following best describes your political preference? (Strongly Democratic/Democratic/Lean Democratic/Strictly Independent/Lean Republican/Republican/Strongly Republican)
- Please select the option that best describes your political orientation. (Very liberal/Somewhat liberal/A bit liberal/Neither liberal nor conservative/A bit conservative/Somewhat conservative/Very conservative)
- On social issues I am: (Strongly liberal/Somewhat liberal/Moderate/Somewhat conservative/Strongly conservative)
- On economic issues I am: (Strongly liberal/Somewhat liberal/Moderate/Somewhat conservative/Strongly conservative)
- Feeling thermometers for Democratic/Republican party voters
- If the 2024 Presidential Election were today, who would you vote for, if anyone? Remember: This survey is anonymous.
- Do you live in an urban, suburban, or rural area?
- Please enter the ZIP code where you live now.

Table S2. Sample composition for Studies 1-5. For categorical variables (gender, education, income, ethnicity, political party, political ideology), we report the number of participants in each category. For age, which is measured as a continuous variable, we report the mean and standard deviation.

Variable		Study 1	Study 2	Study 3	Study 4	Study 5
Gender	Female	486 (49.85%)	481 (50.47%)	485 (50.42%)	482 (51.22%)	503 (50.91%)
	Male	484 (49.64%)	470 (49.32%)	475 (49.38%)	455 (48.35%)	481 (48.68%)
	Other	4 (0.41%)	2 (0.21)	2 (0.21%)	4 (0.43%)	4 (0.4%)
Age	Mean (SD)	44.26 (15.38)	45.27 (15.7)	42.73 (17.4)	40.93 (13.04)	39.29 (12.62)
Education	Less than high school degree	2 (0.21%)	4 (0.42%)	7 (0.73%)	3 (0.32%)	9 (0.91%)
	High school graduate (high school diploma or equivalent including GED)	96 (9.85%)	104 (10.91%)	97 (10.08%)	102 (10.84%)	120 (12.15%)
	Some college but no degree	187 (19.18%)	182 (19.1%)	188 (19.54%)	224 (23.8%)	226 (22.87%)
	Associate degree in college (2-year)	107 (10.97%)	123 (12.91%)	121 (12.58%)	103 (10.95%)	111 (11.23%)
	Bachelor's degree in college (4-year)	419 (42.97%)	393 (41.24%)	370 (38.46%)	371 (39.43%)	364 (36.84%)
	Master's degree	124 (12.72%)	100 (10.49%)	133 (13.83%)	111 (11.8%)	125 (12.65%)
	Doctoral degree	17 (1.74%)	17 (1.78%)	17 (1.77%)	12 (1.28%)	18 (1.82%)
	Professional degree (JD, MD)	23 (2.36%)	30 (3.15%)	29 (3.01%)	15 (1.59%)	15 (1.52%)

Income	Less than $10,000	24 (2.47%)	39 (4.09%)	19 (1.98%)	42 (4.46%)	40 (4.05%)
	$10,000 - $19,999	53 (5.45%)	44 (4.62%)	53 (5.51%)	51 (5.42%)	60 (6.07%)
	$20,000 - $29,999	64 (6.58%)	69 (7.24%)	69 (7.17%)	67 (7.12%)	71 (7.19%)
	$30,000 - $39,999	81 (8.33%)	87 (9.13%)	97 (10.08%)	84 (8.93%)	72 (7.29%)
	$40,000 - $49,999	70 (7.2%)	94 (9.86%)	97 (10.08%)	91 (9.67%)	81 (8.2%)
	$50,000 - $59,999	101 (10.39%)	97 (10.18%)	93 (9.67%)	83 (8.82%)	114 (11.54%)
	$60,000 - $69,999	83 (8.54%)	76 (7.97%)	97 (10.08%)	69 (7.33%)	83 (8.4%)
	$70,000 - $79,999	74 (7.61%)	78 (8.18%)	83 (8.63%)	96 (10.2%)	90 (9.1%)
	$80,000 - $89,999	75 (7.72%)	52 (5.46%)	52 (5.41%)	52 (5.53%)	54 (5.47%)
	$90,000 - $99,999	74 (7.61%)	66 (6.93%)	50 (5.2%)	69 (7.33%)	43 (4.35%)
	$100,000 - $149,999	167 (17.18%)	158 (16.58%)	163 (16.94%)	150 (15.94%)	177 (17.91%)
	$150,000 or more	106 (10.91%)	93 (9.76%)	89 (9.25%)	87 (9.25%)	103 (10.43%)
Ethnicity	White/Caucasian	738 (76.004%)	721 (75.66%)	724 (75.26%)	741 (78.75%)	736 (74.49%)
	Black	130 (13.39%)	123 (12.91%)	132 (13.72%)	101 (10.73%)	134 (13.56%)
	Asian	50 (5.15%)	36 (3.78%)	54 (5.61%)	44 (4.68%)	46 (4.66%)
	Multiracial or other	53 (5.44%)	73 (7.66%)	52 (5.41%)	55 (5.84%)	72 (7.29%)
Political party	Strongly Republican	123 (12.62%)	60 (6.3%)	60 (6.24%)	47 (4.99%)	45 (4.55%)
	Republican	207 (21.23%)	101 (10.6%)	93 (9.67%)	100 (10.63%)	95 (9.62%)
	Lean Republican	132 (13.54%)	115 (12.067%)	100 (10.4%)	115 (12.22%)	120 (12.15%)

	Strictly Independent	31 (3.18%)	165 (17.31%)	181 (18.81%)	198 (21.04%)	183 (18.52%)
	Lean Democratic	106 (10.87%)	185 (19.41%)	159 (16.53%)	174 (18.49%)	176 (17.81%)
	Democratic	188 (19.28%)	150 (15.74%)	220 (22.87%)	175 (18.6%)	204 (20.65%)
	Strongly Democratic	188 (19.28%)	177 (18.57%)	149 (15.49%)	132 (14.03%)	165 (16.7%)
Political ideology	Very conservative	132 (13.59%)	74 (7.76%)	86 (8.94%)	62 (6.59%)	60 (6.07%)
	Somewhat conservative	207 (21.32%)	106 (11.12%)	117 (12.16%)	116 (12.33%)	108 (10.93%)
	A bit conservative	114 (11.74%)	102 (10.7%)	86 (8.94%)	101 (10.73%)	107 (10.83%)
	Neither liberal nor conservative	74 (7.62%)	168 (17.63%)	156 (16.22%)	190 (20.19%)	171 (17.31%)
	A bit liberal	85 (8.75%)	131 (13.75%)	120 (12.47%)	118 (12.54%)	
	Somewhat liberal	189 (19.46%)	207 (21.72%)	241 (25.05%)	198 (21.04%)	221 (22.37%)
	Very liberal	170 (17.51%)	165 (17.31%)	156 (16.22%)	156 (16.58%)	195 (19.74%)

Table S3. Primary analysis for Study 1: OLS regression predicting (log) dwell time and logistic regressions predicting like or not and share or not as a function of positive or negative emotional language with robust standard errors clustered on participants and posts. *p<0.05, **p<0.01, ***p<0.001

	(log) dwell time	like or not	share or not
(Intercept)	1.4 (0.0504)***<br>BF > 100	-2.324 (0.0979)***<br>BF > 100	-2.858 (0.1)***<br>BF > 100
Percent positive emotional language	-0.000293 (0.000843)<br>BF < 0.01	0.00556 (0.0014)***<br>BF = 14.83	0.0004 (0.00136)<br>BF < 0.01
Percent negative emotional language	0.00279 (0.000864)**<br>BF = 1.12	0.000365 (0.00151)<br>BF < 0.01	0.00329 (0.00128)*<br>BF = 0.17
RMSE	1.0557		
Adj. R2	0.00689		
Log-likelihood		-35,612.2	-24,055.3
Adj. Pseudo R2		0.00457	0.00101
BIC		71,259.2	48,145.4
Squared Cor.		0.00317	0.000491

Table S4. Primary analysis for Study 2: OLS regression predicting (log) dwell time and logistic regressions predicting like or not and share or not as a function of the interaction between inoculation treatment and positive or negative emotional language with robust standard errors clustered on participants and posts. *p<0.05, **p<0.01, ***p<0.001

	(log) dwell time	like or not	share or not
(Intercept)	1.133 (0.0453)***<br>BF > 100	-3.406 (0.133)***<br>BF > 100	-4.117 (0.142)***<br>BF > 100
Condition (treatment: control)	-0.0354 (0.0364)<br>BF = 0.01	-0.0537 (0.115)<br>BF < 0.01	-0.04 (0.158)<br>BF < 0.01
Percent positive emotional language	-0.000208 (0.000677)<br>BF < 0.01	0.00748 (0.00206)**<br>BF = 5.67	0.0031 (0.00225)<br>BF = 0.02
Percent negative emotional language	0.00225 (0.000713)**<br>BF = 1.1	-0.000533 (0.00223)<br>BF < 0.01	0.00552 (0.00206)**<br>BF = 0.28
Average pre-treatment dwell time	0.0407 (0.00638)***<br>BF > 100		
Average pre-treatment liking		5.499 (0.384)***<br>BF > 100	
Average pre-treatment sharing			6.153 (0.457)***<br>BF > 100
Number of posts since treatment (centered)	-0.00507 (0.000463)***<br>BF > 100	-0.00669 (0.00179)**<br>BF = 8.11	-0.0113 (0.00282)***<br>BF = 23.57
Condition : percent positive emotional language	-0.000046 (0.000219)<br>BF < 0.01	0.000374 (0.00129)<br>BF <0.01	-0.000025 (0.00169)<br>BF < 0.01
Condition : percent negative emotional language	-0.000068 (0.000255)<br>BF < 0.01	0.000746 (0.00192)<br>BF < 0.01	-0.00203 (0.00172)<br>BF = 0.014
Condition : avg. pre-treatment dwell time	0.0118 (0.0078)<br>BF = 0.02		
Condition : avg. pre-treatment liking		-0.353 (0.597)<br>BF < 0.01	
Condition : avg. pre-treatment sharing			-0.293 (0.701)<br>BF < 0.01
Condition : num. posts since treatment	-0.000433 (0.000647)<br>BF < 0.01	-0.00119 (0.00236)<br>BF < 0.01	0.00176 (0.00372)<br>BF < 0.01
RMSE	0.884		
Adj. R2	0.194		
Log-likelihood		-12,318.0	-7,766.3
Adj. Pseudo R2		0.208	0.266
BIC		24,747.0	15,643.7
Squared Cor.		0.16	0.178

Table S5. Primary analysis for Study 3: OLS regression predicting (log) dwell time and logistic regressions predicting like or not and share or not as a function of the interaction between inoculation treatment and positive or negative emotional language with robust standard errors clustered on participants and posts. *p<0.05, **p<0.01, ***p<0.001

	(log) dwell time	like or not	share or not
(Intercept)	1.473 (0.0554)***<br>BF > 100	-2.792 (0.136)***<br>BF > 100	-3.584 (0.154)***<br>BF > 100
Condition (treatment: control)	-0.0122 (0.0416)<br>BF < 0.01	-0.0319 (0.0914)<br>BF < 0.01	-0.065 (0.143)<br>BF < 0.01
Percent positive emotional language	0.000186 (0.000922)<br>BF < 0.01	0.00663 (0.00207)**<br>BF = 1.55	0.000645 (0.00236)<br>BF < 0.01
Percent negative emotional language	0.00332 (0.000904)**<br>BF = 7.58	0.000516 (0.00222)<br>BF < 0.01	0.00536 (0.00225)*<br>BF = 0.15
Average pre-treatment dwell time	0.0219 (0.00753)**<br>BF = 0.63		
Average pre-treatment liking		4.988 (0.291)***<br>BF > 100	
Average pre-treatment sharing			5.344 (0.408)***<br>BF > 100
Number of posts since treatment (centered)	-0.0058 (0.000594)***<br>BF > 100	-0.00749 (0.00171)***<br>BF > 100	-0.00307 (0.00244)<br>BF = 0.02
Condition : percent positive emotional language	-0.000616 (0.000289)*<br>BF = 0.086	0.00147 (0.00101)<br>BF = 0.02	0.00125 (0.00186)<br>BF < 0.01
Condition : percent negative emotional language	-0.000433 (0.000287)<br>BF = 0.026	-0.00148 (0.00138)<br>BF = 0.014	0.00122 (0.00181)<br>BF < 0.01
Condition : avg. pre-treatment dwell time	0.0186 (0.0105)<br>BF = 0.042		
Condition : avg. pre-treatment liking		-0.683 (0.363)<br>BF = 0.051	
Condition : avg. pre-treatment sharing			0.0599 (0.54)<br>BF < 0.01
Condition : num. posts since treatment	0.000396 (0.000781)<br>BF < 0.01	-0.00349 (0.00233)<br>BF = 0.026	-0.00337 (0.00337)<br>BF = 0.012
RMSE	0.96		
Adj. R2	0.148		
Log-likelihood		-13,065.6	-8,069.9
Adj. Pseudo R2		0.184	0.231
BIC		26,239.1	16,247.9
Squared Cor.		0.165	0.157

Table S6. Primary analysis for Study 4: OLS regression predicting (log) dwell time and logistic regressions predicting like or not and share or not as a function of the interaction between inoculation treatment and the presence of emotional manipulation with robust standard errors clustered on participants and posts. *p<0.05, **p<0.01, ***p<0.001

	(log) dwell time	like or not	share or not
(Intercept)	2.397 (0.0394)***<br>BF > 100	-2.471 (0.228)***<br>BF > 100	-2.521 (0.179)***<br>BF > 100
Condition (treatment: control)	-0.0493 (0.0566)<br>BF = 0.018	0.163 (0.127)<br>BF = 0.031	-0.12 (0.144)<br>BF = 0.016
Manipulative or not (manipulative : neutral)	0.000232 (0.0109)<br>BF < 0.01	-0.101 (0.227)<br>BF = 0.011	0.078 (0.193)<br>BF = 0.01
Average pre-treatment dwell time	0.0219 (0.00323)***<br>BF > 100		
Average pre-treatment liking		5.17 (0.347)***<br>BF > 100	
Average pre-treatment sharing			5.0041 (0.334)***<br>BF > 100
Number of posts since treatment (centered)	-0.00585 (0.00093)***<br>BF > 100	-0.01 (0.00356)**<br>BF = 1.15	-0.00606 (0.0021)**<br>BF = 1.04
Condition : manipulative or not	0.00304 (0.00312)<br>BF = 0.02	-0.192 (0.101)<br>BF = 0.09	-0.088 (0.106)<br>BF = 0.017
Condition : avg. pre-treatment dwell time	-0.00644 (0.00482)<br>BF = 0.034		
Condition : avg. pre-treatment liking		-0.597 (0.396)<br>BF = 0.045	
Condition : avg. pre-treatment sharing			-0.0694 (0.489)<br>BF < 0.01
Condition : num. posts since treatment	-0.00174 (0.00139)<br>BF = 0.03	0.00307 (0.00436)<br>BF = 0.015	-0.0017 (0.00399)<br>BF = 0.01
RMSE	1.09		
Adj. R2	0.161		
Log-likelihood		-4,844.6	-4,613.9
Adj. Pseudo R2		0.25	0.202
BIC		9,767.1	9,305.7
Squared Cor.		0.247	0.177

Table S7. Primary analysis for Study 4: OLS regression predicting (log) dwell time and logistic regressions predicting like or not and share or not as a function of the interaction between inoculation treatment and positive or negative emotional language with robust standard errors clustered on participants and posts. *p<0.05, **p<0.01, ***p<0.001

	(log) dwell time	like or not	share or not
(Intercept)	2.459 (0.0397)***<br>BF > 100	-2.423 (0.227)***<br>BF > 100	-2.294 (0.302)***<br>BF > 100
Condition (treatment: control)	-0.0371 (0.0501)<br>BF = 0.015	0.208 (0.141)<br>BF = 0.04	-0.24 (0.178)<br>BF = 0.035
Percent positive emotional language	-0.000973 (0.00016)***<br>BF > 100	0.00559 (0.00339)<br>BF = 0.06	-0.0039 (0.00353)<br>BF = 0.024
Percent negative emotional language	-0.000475 (0.000134)***<br>BF = 8.41	-0.00378 (0.00298)<br>BF = 0.03	-0.00233 (0.00355)<br>BF = 0.014
Average pre-treatment dwell time	0.0231 (0.00328)***<br>BF > 100		
Average pre-treatment liking		5.236 (0.338)***<br>BF > 100	
Average pre-treatment sharing			5.011 (0.332)***<br>BF > 100
Number of posts since treatment (centered)	-0.00645 (0.000962)***<br>BF > 100	-0.0109 (0.00365)**<br>BF = 1.3	-0.00599 (0.00207)**<br>BF = 0.99
Condition : percent positive emotional language	-0.000096 (0.00026)<br>BF = 0.01	-0.00419 (0.00154)**<br>BF = 0.62	0.00217 (0.00264)<br>BF = 0.017
Condition : percent negative emotional language	-0.000095 (0.000243)<br>BF = 0.01	-0.000927 (0.0016)<br>BF = 0.01	0.001 (0.00192)<br>BF = 0.012
Condition : avg. pre-treatment dwell time	-0.00615 (0.00501)<br>BF = 0.03		
Condition : avg. pre-treatment liking		-0.645 (0.398)<br>BF = 0.054	
Condition : avg. pre-treatment sharing			-0.0748 (0.49)<br>BF < 0.01
Condition : num. posts since treatment	-0.00156 (0.00141)<br>BF = 0.025	0.00332 (0.00435)<br>BF = 0.016	-0.00175 (0.00396)<br>BF = 0.01
RMSE	1.116		
Adj. R2	0.174		
Log-likelihood		-4,808.5	-4,611.4
Adj. Pseudo R2		0.255	0.202
BIC		9,714.4	9,320.2
Squared Cor.		0.25	0.178

Table S8. Primary analysis for Study 5: OLS regression predicting (log) dwell time and logistic regressions predicting like or not and share or not as a function of the interaction between inoculation treatment and the presence of emotional manipulation with robust standard errors clustered on participants and posts. *p<0.05, **p<0.01, ***p<0.001

	(log) dwell time	like or not	share or not
(Intercept)	2.481 (0.0411)***<br>BF > 100	-2.298 (0.227)***<br>BF > 100	-2.536 (0.163)***<br>BF > 100
Condition (treatment: control)	0.00558 (0.0597)<br>BF < 0.01	0.0181 (0.102)<br>BF < 0.01	0.048 (0.113)<br>BF = 0.01
Manipulative or not (manipulative : neutral)	-0.0149 (0.00184)***<br>BF > 100	-0.192 (0.231)<br>BF = 0.016	0.263 (0.194)<br>BF = 0.032
Average pre-treatment dwell time	0.00543 (0.00503)<br>BF = 0.02		
Average pre-treatment liking		4.282 (0.318)***<br>BF > 100	
Average pre-treatment sharing			4.87 (0.36)***<br>BF > 100
Number of posts since treatment (centered)	-0.0138 (0.00269)***<br>BF > 100	-0.00919 (0.00637)<br>BF = 0.037	0.00699 (0.00494)<br>BF = 0.036
Condition : manipulative or not	-0.000244 (0.0016)<br>BF < 0.01	0.0505 (0.0851)<br>BF = 0.01	-0.217 (0.0622)***<br>BF = 6.05
Condition : avg. pre-treatment dwell time	0.0192 (0.00841)*<br>BF = 0.2		
Condition : avg. pre-treatment liking		0.798 (0.333)*<br>BF = 0.25	
Condition : avg. pre-treatment sharing			0.149 (0.439)<br>BF < 0.01
Condition : num. posts since treatment	-0.000623 (0.003)<br>BF < 0.01	-0.00598 (0.00949)<br>BF = 0.013	-0.0126 (0.00712)<br>BF = 0.065
RMSE	1.0528		
Adj. R2	0.132		
Log-likelihood		-6,094.6	-5,755.2
Adj. Pseudo R2		0.22	0.207
BIC		12,268.4	11,589.6
Squared Cor.		0.216	0.189

Table S9. Primary analysis for Study 5: OLS regression predicting (log) dwell time and logistic regressions predicting like or not and share or not as a function of the interaction between inoculation treatment and positive or negative emotional language with robust standard errors clustered on participants and posts. *p<0.05, **p<0.01, ***p<0.001

	(log) dwell time	like or not	share or not
(Intercept)	2.532 (0.0438)***<br>BF > 100	-2.323 (0.266)***<br>BF > 100	-2.225 (0.19)***<br>BF > 100
Condition (treatment: control)	-0.0312 (0.0591)<br>BF = 0.01	0.134 (0.127)<br>BF = 0.02	-0.00162 (0.107)<br>BF < 0.01
Percent positive emotional language	-0.000744 (0.000118)***<br>BF > 100	0.00596 (0.00409)<br>BF = 0.04	-0.00401 (0.00202)*<br>BF = 0.099
Percent negative emotional language	-0.000496 (0.000075)***<br>BF > 100	-0.00329 (0.00351)<br>BF = 0.018	-0.00208 (0.00269)<br>BF = 0.015
Average pre-treatment dwell time	0.0058 (0.00531)<br>BF = 0.022		
Average pre-treatment liking		4.333 (0.309)***<br>BF > 100	
Average pre-treatment sharing			4.858 (0.36)***<br>BF > 100
Number of posts since treatment (centered)	-0.0154 (0.0024)***<br>BF > 100	-0.00942 (0.00655)<br>BF = 0.037	0.00636 (0.00485)<br>BF = 0.03
Condition : percent positive emotional language	0.000965 (0.000392)*<br>BF = 0.3	-0.00266 (0.00165)<br>BF = 0.05	-0.00103 (0.000894)<br>BF = 0.024
Condition : percent negative emotional language	0.000549 (0.000184)**<br>BF = 1.19	-0.000732 (0.00123)<br>BF = 0.01	-0.000919 (0.00101)<br>BF = 0.018
Condition : avg. pre-treatment dwell time	0.0201 (0.00882)*<br>BF = 0.19		
Condition : avg. pre-treatment liking		0.785 (0.337)*<br>BF = 0.2	
Condition : avg. pre-treatment sharing			0.165 (0.439)<br>BF < 0.01
Condition : num. posts since treatment	-0.000366 (0.00324)<br>BF < 0.01	-0.00594 (0.00957)<br>BF = 0.013	-0.0118 (0.00713)<br>BF = 0.054
RMSE	1.0885		
Adj. R2	0.137		
Log-likelihood		-6,037.9	-5,753.7
Adj. Pseudo R2		0.227	0.207
BIC		12,174.7	11,606.3
Squared Cor.		0.218	0.19

Table S10. Study 2 robustness check: remove participants who failed attention check in Qualtrics. *p<0.05, **p<0.01, ***p<0.001

	(log) dwell time	like or not	share or not
(Intercept)	1.134 (0.0455) ***	-3.434 (0.134) ***	-4.121 (0.141) ***
Condition (treatment: control)	-0.0338 (0.0365)	-0.0393 (0.114)	-0.0839 (0.159)
Percent positive emotional language	-0.000200 (0.000679)	0.00765 (0.00209) ***	0.00320 (0.00225)
Percent negative emotional language	0.00228 (0.000716) **	-0.000394 (0.00225)	0.00572 (0.00205) **
Average pre-treatment dwell time	0.0407 (0.00639) ***		
Average pre-treatment liking		5.765 (0.319) ***	
Average pre-treatment sharing			-0.00112 (0.00284) ***
Number of posts since treatment (centered)	-0.00506 (0.000466) ***	-0.00634 (0.00179) ***	-0.0112 (0.00284) ***
Condition : percent positive emotional language	-0.000013 (0.000215)	0.000192 (0.00132)	-0.000100 (0.00176)
Condition : percent negative emotional language	-0.000042 (0.000255)	0.000875 (0.00192)	-0.00192 (0.00178)
Condition : avg. pre-treatment dwell time	0.0122 (0.00783)		
Condition : avg. pre-treatment liking		-0.641 (0.558)	
Condition : avg. pre-treatment sharing			0.156 (0.613)
Condition : num. posts since treatment	-0.00053 (0.00066)	-0.00166 (0.00238)	0.00139 (0.00377)
RMSE	0.884		
Adj. R2	0.196		
Log-likelihood		-12048.8	-7597.9
Adj. Pseudo R2		0.214	0.276
BIC		24208.6	15306.7
Squared Cor.		0.168	0.193

Table S11. Study 3 robustness check: remove participants who failed attention check in Qualtrics. *p<0.05, **p<0.01, ***p<0.001

	(log) dwell time	like or not	share or not
(Intercept)	1.476 (0.00556) ***	-2.803 (0.137) ***	-3.59 (0.157) ***
Condition (treatment: control)	-0.00636 (0.0419)	-0.0173 (0.0937)	-0.0656 (0.146)
Percent positive emotional language	0.000167 (0.000921)	0.00678 (0.00208) **	0.000659 (0.00239)
Percent negative emotional language	0.00334 (0.000906) ***	0.000540 (0.00224)	0.00558 (0.00229) *
Average pre-treatment dwell time	0.0217 (0.0.00751) **		
Average pre-treatment liking		4.964 (0.295) ***	
Average pre-treatment sharing			5.294 (0.407) ***
Number of posts since treatment (centered)	-0.00586 (0.000602) ***	-0.00784 (0.00174) ***	-0.00309 (0.00252)
Condition : percent positive emotional language	-0.000637 (0.000290) *	0.00126 (0.00106)	0.00113 (0.00194)
Condition : percent negative emotional language	-0.000482 (0.000292)	-0.00125 (0.00140)	0.000723 (0.00184)
Condition : avg. pre-treatment dwell time	0.0182 (0.0105) .		
Condition : avg. pre-treatment liking		-0.6560 (0.373) .	
Condition : avg. pre-treatment sharing			0.310 (0.513)
Condition : num. posts since treatment	0.000443 (0.000787)	-0.00302 (0.00235)	-0.00335 (0.00349)
RMSE	0.961		
Adj. R2	0.146		
Log-likelihood		-12777.5	-7837.8
Adj. Pseudo R2		0.182	0.230
BIC		25662.7	15783.3
Squared Cor.		0.162	0.159

Table S12. Study 4 robustness check: remove participants who failed attention check in Qualtrics. Manipulative or neutral dummy variable. *p<0.05, **p<0.01, ***p<0.001

	(log) dwell time	like or not	share or not
(Intercept)	2.405 (0.0397) ***	-2.452 (0.223) ***	-2.546 (0.186) ***
Condition (treatment: control)	-0.0605 (0.0575)	0.124 (0.125)	-0.0981 (0.147)
Manipulative or not (manipulative : neutral)	-0.00584 (0.0106)	-0.116 (0.227)	0.0917 (0.201)
Average pre-treatment dwell time	0.0218 (0.00326) ***		
Average pre-treatment liking		5.187 (0.356) ***	
Average pre-treatment sharing			4.970 (0.336) ***
Number of posts since treatment (centered)	-0.00558 (0.000948) ***	-0.0101 (0.00349) **	-0.00570 (0.00232) *
Condition : manipulative or not	0.00861 (0.00319) **	-0.167 (0.0974) .	-0.0807 (0.110)
Condition : avg. pre-treatment dwell time	-0.00647 (0.00483)		
Condition : avg. pre-treatment liking		-0.494 (0.407)	
Condition : avg. pre-treatment sharing			0.0179 (0.510)
Condition : num. posts since treatment	-0.00230 (0.00143)	0.00210 (0.00437)	-0.00157 (0.00411)
RMSE	1.090		
Adj. R2	0.162		
Log-likelihood		-4635.3	-4431.5
Adj. Pseudo R2		0.246	0.199
BIC		9348.3	8940.7
Squared Cor.		0.241	0.173

Table S13. Study 4 robustness check: remove participants who failed attention check in Qualtrics. Percent emotional language variables. *p<0.05, **p<0.01, ***p<0.001

	(log) dwell time	like or not	share or not
(Intercept)	2.468 (0.0396) ***	-2.383 (0.222) ***	-2.332 (0.323) ***
Condition (treatment: control)	-0.0481 (0.0511)	0.146 (0.134)	-0.216 (0.208)
Percent positive emotional language	-0.00105 (0.000125) ***	0.00528 (0.00333)	-0.00330 (0.00377)
Percent negative emotional language	-0.000526 (0.000109) ***	-0.00420 (0.00297)	-0.00214 (0.00383)
Average pre-treatment dwell time	0.0230 (0.00330) ***		
Average pre-treatment liking		5.256 (0.348) ***	
Average pre-treatment sharing			4.976 (0.334) ***
Number of posts since treatment (centered)	-0.00620 (0.0000982) ***	-0.0105 (0.00358) **	-0.00564 (0.00233) *
Condition : percent positive emotional language	-0.00092 (0.000263)	-0.00384 (0.00143) **	0.00147 (0.00290)
Condition : percent negative emotional language	-0.000052 (0.000242)	-0.000379 (0.00145)	0.00101 (0.00231)
Condition : avg. pre-treatment dwell time	-0.00618 (0.00502)		
Condition : avg. pre-treatment liking		-0.542 (0.410)	
Condition : avg. pre-treatment sharing			0.133 (0.510)
Condition : num. posts since treatment	-0.00210 (0.00145)	0.00234 (0.00439)	-0.00161 (0.00408)
RMSE	1.117		
Adj. R2	0.175		
Log-likelihood		-4599.9	-4429.9
Adj. Pseudo R2		0.252	0.199
BIC		9297.0	8957.0
Squared Cor.		0.243	0.173

Table S14. Study 5 robustness check: remove participants who failed attention check in Qualtrics. Manipulative or neutral dummy variable. *p<0.05, **p<0.01, ***p<0.001

	(log) dwell time	like or not	share or not
(Intercept)	2.497 (0.0419) ***	-2.302 (0.230) ***	-2.546 (0.162) ***
Condition (treatment: control)	-0.00528 (0.0599)	0.0227 (0.0997)	0.0541 (0.113)
Manipulative or not (manipulative : neutral)	-0.0188 (0.00156) ***	-0.189 (0.239)	0.257 (0.192)
Average pre-treatment dwell time	0.00520 (0.00493)		
Average pre-treatment liking		4.224 (0.318) ***	
Average pre-treatment sharing			4.800 (0.368) ***
Number of posts since treatment (centered)	-0.0137 (0.00267) ***	-0.00824 (0.00700)	0.00521 (0.00536)
Condition : manipulative or not	0.00285 (0.00141) *	0.0423 (0.0831)	-0.207 (0.0553) ***
Condition : avg. pre-treatment dwell time	0.0190 (0.00831) *		
Condition : avg. pre-treatment liking		0.822 (0.345) *	
Condition : avg. pre-treatment sharing			0.212 (0.446)
Condition : num. posts since treatment	-0.000556 (0.00300)	-0.00373 (0.00963)	-0.0107 (0.00706)
RMSE	1.050		
Adj. R2	0.130		
Log-likelihood		-5878.8	-5566.8
Adj. Pseudo R2		0.214	0.207
BIC		11836.5	11212.5
Squared Cor.		0.208	0.189

Table S15. Study 5 robustness check: remove participants who failed attention check in Qualtrics. Percent emotional language variables. *p<0.05, **p<0.01, ***p<0.001

	(log) dwell time	like or not	share or not
(Intercept)	2.551 (0.0448) ***	-2.317 (0.270) ***	-2.241 (0.188) ***
Condition (treatment: control)	-0.0417 (0.0582)	0.119 (0.119)	0.0235 (0.104)
Percent positive emotional language	-0.000814 (0.000132) ***	0.00608 (0.00411)	-0.00433 (0.00198) *
Percent negative emotional language	-0.000577 (0.000073) ***	-0.00351 (0.00360)	-0.00195 (0.00264)
Average pre-treatment dwell time	0.00551 (0.00517)		
Average pre-treatment liking		4.278 (0.309) ***	
Average pre-treatment sharing			4.795 (0.367) ***
Number of posts since treatment (centered)	-0.152 (0.00251) ***	-0.00871 (0.00715)	0.00472 (0.00526)
Condition : percent positive emotional language	0.00100 (0.000417) *	-0.00243 (0.00163)	-0.00103 (0.000843)
Condition : percent negative emotional language	0.000592 (0.000219) **	-0.000503 (0.00113)	-0.00117 (0.000865)
Condition : avg. pre-treatment dwell time	0.0199 (0.00870) *		
Condition : avg. pre-treatment liking		-0.809 (0.350) *	
Condition : avg. pre-treatment sharing			0.227 (0.446)
Condition : num. posts since treatment	-0.000404 (0.00328)	-0.00346 (0.00968)	-0.010 (0.00705)
RMSE	1.0853		
Adj. R2	0.134		
Log-likelihood		-5819.2	-5563.7
Adj. Pseudo R2		0.222	0.207
BIC		11737.0	11226.0
Squared Cor.		0.211	0.190

Table S16. Study 2 robustness check: remove participants who were inactive at all during video pop-up. *p<0.05, **p<0.01, ***p<0.001

	(log) dwell time	like or not	share or not
(Intercept)	1.185 (0.0492) ***	-3.420 (0.144) ***	-4.0353 (0.154) ***
Condition (treatment: control)	-0.0556 (0.0410)	0.00311 (0.141)	-0.224 (0.193)
Percent positive emotional language	-0.000295 (0.000742)	0.00774 (0.00215) ***	0.002267 (0.00226)
Percent negative emotional language	0.00247 (0.000768) **	0.000368 (0.00227)	0.00585 (0.00213) **
Average pre-treatment dwell time	0.0475 (0.00495) ***		
Average pre-treatment liking		5.573 (0.431) ***	
Average pre-treatment sharing			6.0753 (0.539) ***
Number of posts since treatment (centered)	-0.00542 (0.000547) ***	-0.00748 (0.00220) ***	-0.00918 (0.00276) ***
Condition : percent positive emotional language	0.000146 (0.000244)	0.000166 (0.00157)	0.000644 (0.00216)
Condition : percent negative emotional language	-0.00000541 (0.000277)	0.000333 (0.00234)	-0.000940 (0.00226)
Condition : avg. pre-treatment dwell time	0.00226 (0.00731)		
Condition : avg. pre-treatment liking		-0.273 (0.756)	
Condition : avg. pre-treatment sharing			0.195 (0.741)
Condition : num. posts since treatment	-0.000604 (0.000778)	0.00099 (0.00279)	-0.00170 (0.00409)
RMSE	0.883		
Adj. R2	0.199		
Log-likelihood		-9203.2	-5705.8
Adj. Pseudo R2		0.221	0.268
BIC		18514.3	11519.5
Squared Cor.		0.174	0.178

Table S17. Study 2 robustness check: remove participants who unmuted the video after the video ended (>108s). · p=0.05, *p<0.05, **p<0.01, ***p<0.001

	(log) dwell time	like or not	share or not
(Intercept)	1.201 (0.00555) ***	-3.262 (0.153) ***	-4.040 (0.158) ***
Condition (treatment: control)	-0.0942 (0.0565) ·	-0.134 (0.174)	-0.364 (0.238)
Percent positive emotional language	-0.000041 (0.000767)	0.00621 (0.00235) **	0.00208 (0.00264)
Percent negative emotional language	0.00270 (0.000820) **	-0.00136 (0.00245)	0.00477 (0.00238) *
Average pre-treatment dwell time	0.00337 (0.00810) ***		
Average pre-treatment liking		5.5621 (0.429) ***	
Average pre-treatment sharing			7.070 (0.402) ***
Number of posts since treatment (centered)	-0.00602 (0.000670) ***	-0.00523 (0.00231) **	-0.0102 (0.00344) **
Condition : percent positive emotional language	-0.000381 (0.000324)	0.00132 (0.00179)	0.000861 (0.00296)
Condition : percent negative emotional language	-0.000402 (0.000430)	0.000200 (0.00263)	-0.000104 (0.00235)
Condition : avg. pre-treatment dwell time	0.0182 (0.0104) ·		
Condition : avg. pre-treatment liking		0.465 (1.080)	
Condition : avg. pre-treatment sharing			-0.336 (0.587)
Condition : num. posts since treatment	0.00118 (0.00101)	0.00204 (0.00327)	-0.00111 (0.00533)
RMSE	0.901		
Adj. R2	0.204		
Log-likelihood		-5670.5	-3440.8
Adj. Pseudo R2		0.222	0.317
BIC		11443.0	6983.8
Squared Cor.		0.189	0.260

Table S18. Study 3 robustness check: remove participants who were inactive at all during video pop-up. *p<0.05, **p<0.01, ***p<0.001

	(log) dwell time	like or not	share or not
(Intercept)	1.545 (0.0570) ***	-2.755 (0.141) ***	-3.490 (0.168) ***
Condition (treatment: control)	-0.0114 (0.0432)	-0.0201 (0.107)	-0.107 (0.164)
Percent positive emotional language	0.00094 (0.000975)	0.00686 (0.00214) **	0.0000918 (0.00247)
Percent negative emotional language	0.00319 (0.000926) ***	0.000599 (0.00241)	0.00669 (0.00243) **
Average pre-treatment dwell time	0.0365 (0.00478) ***		
Average pre-treatment liking		4.987 (0.299) ***	
Average pre-treatment sharing			5.589 (0.412) ***
Number of posts since treatment (centered)	-0.000630 (0.000683) ***	-0.00903 (0.00191) ***	-0.00406 (0.00287)
Condition : percent positive emotional language	-0.000388 (0.000314)	0.000627 (0.00123)	0.00127 (0.00220)
Condition : percent negative emotional language	-0.000144 (0.000340)	-0.00192 (0.00156)	0.000060 (0.00228)
Condition : avg. pre-treatment dwell time	0.00338 (0.0109)		
Condition : avg. pre-treatment liking		-0.782 (0.382) *	
Condition : avg. pre-treatment sharing			-0.313 (0.573)
Condition : num. posts since treatment	0.000709 (0.000895)	-0.00111 (0.00255)	-0.00372 (0.00429)
RMSE	0.928		
Adj. R2	0.185		
Log-likelihood		-9526.5	-5783.9
Adj. Pseudo R2		0.166	0.233
BIC		19157.7	11672.5
Squared Cor.		0.142	0.171

Table S19. Study 4 robustness check: remove participants who were inactive at all during video pop-up. Manipulative or neutral dummy variable. *p<0.05, **p<0.01, ***p<0.001

	(log) dwell time	like or not	share or not
(Intercept)	2.500 (0.0419) ***	-2.458 (0.234) ***	-2.483 (0.187) ***
Condition (treatment: control)	-0.0336 (0.0610)	0.198 (0.151)	0.00186 (0.162)
Manipulative or not (manipulative : neutral)	0.00127 (0.0133)	-0.0914 (0.225)	0.0827 (0.202)
Average pre-treatment dwell time	0.00228 (0.00424) ***		
Average pre-treatment liking		5.145 (0.375) ***	
Average pre-treatment sharing			5.0364 (0.364) ***
Number of posts since treatment (centered)	-0.00616 (0.00125) ***	-0.114 (0.00335) **	-0.00338 (0.00243)
Condition : manipulative or not	-0.00611 (0.00334) .	-0.114 (0.110)	-0.0836 (0.110)
Condition : avg. pre-treatment dwell time	-0.00705 (0.00590)		
Condition : avg. pre-treatment liking		-0.946 (0.432) *	
Condition : avg. pre-treatment sharing			-0.309 (0.575)
Condition : num. posts since treatment	-0.00120 (0.00154)	-0.000570 (0.00460)	-0.00343 (0.00388)
RMSE	1.0379		
Adj. R2	0.167		
Log-likelihood		-3773.1	-3658.3
Adj. Pseudo R2		0.233	0.205
BIC		7621.7	7392.1
Squared Cor.		0.232	0.191

Table S20. Study 4 robustness check: remove participants who were inactive at all during video pop-up. Percent emotional language variables. *p<0.05, **p<0.01, ***p<0.001

	(log) dwell time	like or not	share or not
(Intercept)	2.560 (0.0503) ***	-2.384 (0.263) ***	-2.253 (0.320) ***
Condition (treatment: control)	-0.0247 (0.0586)	0.129 (0.159)	-0.0838 (0.215)
Percent positive emotional language	-0.000888 (0.000344) **	0.00513 (0.00359)	-0.00451 (0.00378)
Percent negative emotional language	-0.000479 (0.000384)	-0.00401 (0.00334)	-0.00223 (0.00376)
Average pre-treatment dwell time	0.0240 (0.00437) ***		
Average pre-treatment liking		5.212 (0.369) ***	
Average pre-treatment sharing			5.045 (0.362) ***
Number of posts since treatment (centered)	-0.00675 (0.00112) ***	-0.0103 (0.00349) **	-0.00325 (0.00242)
Condition : percent positive emotional language	-0.000308 (0.000349)	-0.00231 (0.00168)	0.00232 (0.00296)
Condition : percent negative emotional language	-0.000070 (0.000376)	0.00127 (0.00170)	0.000219 (0.00224)
Condition : avg. pre-treatment dwell time	-0.00624 (0.00612)		
Condition : avg. pre-treatment liking		-0.995 (0.434) *	
Condition : avg. pre-treatment sharing			-0.315 (0.575)
Condition : num. posts since treatment	-0.000944 (0.00151)	-0.000325 (0.00464)	-0.00352 (0.00392)
RMSE	1.061		
Adj. R2	0.184		
Log-likelihood		-3745.5	-3655.2
Adj. Pseudo R2		0.238	0.206
BIC		7585.4	7404.9
Squared Cor.		0.234	0.192

Table S21. Study 5 robustness check: remove participants who were inactive at all during video pop-up. Manipulative or neutral dummy variable. *p<0.05, **p<0.01, ***p<0.001

	(log) dwell time	like or not	share or not
(Intercept)	2.581 (0.0456) ***	-2.274 (0.237) ***	-2.443 (0.165) ***
Condition (treatment: control)	-0.0133 (0.0608)	0.0762 (0.0955)	0.0266 (0.132)
Manipulative or not (manipulative : neutral)	-0.0123 (0.00531) *	-0.156 (0.254)	0.261 (0.201)
Average pre-treatment dwell time	0.00345 (0.00399)		
Average pre-treatment liking		4.043 (0.318) ***	
Average pre-treatment sharing			4.589 (0.374) ***
Number of posts since treatment (centered)	-0.0150 (0.00253) ***	-0.00857 (0.00692)	0.0105 (0.00559) .
Condition : manipulative or not	-0.00956 (0.00485) *	-0.0101 (0.0822)	-0.233 (0.0713) **
Condition : avg. pre-treatment dwell time	0.0266 (0.00534) ***		
Condition : avg. pre-treatment liking		0.762 (0.384) *	
Condition : avg. pre-treatment sharing			0.756 (0.480)
Condition : num. posts since treatment	-0.000213 (0.00304)	-0.00941 (0.0101)	-0.0166 (0.00918) .
RMSE	0.984		
Adj. R2	0.153		
Log-likelihood		-4641.5	-4419.2
Adj. Pseudo R2		0.194	0.203
BIC		9359.8	8915.1
Squared Cor.		0.182	0.189

Table S22. Study 5 robustness check: remove participants who were inactive at all during video pop-up. Percent emotional language variables. *p<0.05, **p<0.01, ***p<0.001

	(log) dwell time	like or not	share or not
(Intercept)	2.639 (0.0478) ***	-2.230 (0.295) ***	-2.123 (0.195) ***
Condition (treatment: control)	-0.0675 (0.0568)	0.162 (0.116)	-0.0382 (0.129)
Percent positive emotional language	-0.000889 (0.000155) ***	0.00655 (0.00441)	-0.00403 (0.00210) .
Percent negative emotional language	-0.000593 (0.000140) ***	-0.00318 (0.00392)	-0.00224 (0.00278)
Average pre-treatment dwell time	0.00379 (0.00427)		
Average pre-treatment liking		4.0999 (0.314) ***	
Average pre-treatment sharing			4.583 (0.375) ***
Number of posts since treatment (centered)	-0.0164 (0.0273) ***	-0.00897 (0.00697)	0.0102 (0.00558) .
Condition : percent positive emotional language	0.000929 (0.000190) ***	-0.00204 (0.00159)	-0.000870 (0.00120)
Condition : percent negative emotional language	0.000779 (0.000136) ***	-0.000922 (0.00120)	-0.000836 (0.000925)
Condition : avg. pre-treatment dwell time	0.0283 (0.00548) ***		
Condition : avg. pre-treatment liking		0.755 (0.387)	
Condition : avg. pre-treatment sharing			0.772 (0.480)
Condition : num. posts since treatment	0.000213 (0.00331)	-0.00951 (0.00995)	-0.0160 (0.00915) .
RMSE	1.0112		
Adj. R2	0.164		
Log-likelihood		-4586.6	-4418.3
Adj. Pseudo R2		0.203	0.203
BIC		9269.1	8932.5
Squared Cor.		0.186	0.190

Table S23. Study 4: regression per outcome variable. Incoherence manipulation technique. *p<0.05, **p<0.01, ***p<0.001

	(log) dwell time	like or not	share or not
(Intercept)	2.381 (0.0435) ***	-2.0285 (0.187) ***	-2.995 (0.162) ***
Condition (treatment: control)	-0.0629 (0.0666)	0.0893 (0.158)	0.0216 (0.178)
Manipulative or not (manipulative : neutral)	0.0262 (0.0241)	-0.642 (0.218) **	-0.404 (0.186) *
Average pre-treatment dwell time	0.0220 (0.00311) ***		
Average pre-treatment liking		5.401 (0.450) ***	
Average pre-treatment sharing			5.229 (0.429) ***
Number of posts since treatment (centered)	-0.00696 (0.00131) ***	-0.00579 (0.00361)	-0.0181 (0.00862) *
Condition : manipulative or not	-0.0263 (0.0342)	-0.0657 (0.171)	-0.252 (0.223)
Condition : avg. pre-treatment dwell time	-0.00670 (0.00472)		
Condition : avg. pre-treatment liking		-0.831 (0.518)	
Condition : avg. pre-treatment sharing			-0.187 (0.654)
Condition : num. posts since treatment	-0.000779 (0.00161)	-0.00625 (0.00559)	0.0218 (0.0125) .
RMSE	1.0951		
Adj. R2	0.162		
Log-likelihood		-2157.9	-1329.9
Adj. Pseudo R2		0.257	0.227
BIC		4386.7	2730.7
Squared Cor.		0.263	0.161

Table S24. Study 4: regression per outcome variable. False dichotomies manipulation technique. *p<0.05, **p<0.01, ***p<0.001

	(log) dwell time	like or not	share or not
(Intercept)	2.401 (0.0384) ***	-1.875 (0.203) ***	-2.853 (0.154) ***
Condition (treatment: control)	-0.0742 (0.0644)	0.0795 (0.137)	-0.164 (0.152)
Manipulative or not (manipulative : neutral)	-0.0396 (0.00760) ***	-0.142 (0.274)	-0.135 (0.251)
Average pre-treatment dwell time	0.00224 (0.00317) ***		
Average pre-treatment liking		4.574 (0.336) ***	
Average pre-treatment sharing			4.862 (0.316) ***
Number of posts since treatment (centered)	-0.00895 (0.000981) ***	-0.00823 (0.00249) ***	-0.0126 (0.00520) *
Condition : manipulative or not	-0.00648 (0.00484)	-0.341 (0.146) *	-0.0966 (0.155)
Condition : avg. pre-treatment dwell time	-0.00648 (0.00484)		
Condition : avg. pre-treatment liking		0.0306 (0.428)	
Condition : avg. pre-treatment sharing			0.931 (0.587)
Condition : num. posts since treatment	0.00137 (0.00180)	-0.00648 (0.00531)	0.00727 (0.00682)
RMSE	1.0971		
Adj. R2	0.0161		
Log-likelihood		-2623.7	-1591.0
Adj. Pseudo R2		0.222	0.231
BIC		5318.7	3253.4
Squared Cor.		0.233	0.177

Table S25. Study 4: regression per outcome variable. Scapegoating manipulation technique. *p<0.05, **p<0.01, ***p<0.001

	(log) dwell time	like or not	share or not
(Intercept)	2.416 (0.0411) ***	-2.359 (0.285) ***	-3.231 (0.157) ***
Condition (treatment: control)	-0.0792 (0.0663)	0.326 (0.176) .	0.305 (0.171) .
Manipulative or not (manipulative : neutral)	-0.0297 (0.00677) ***	-0.357 (0.290)	-0.0779 (0.148)
Average pre-treatment dwell time	0.0233 (0.00334) ***		
Average pre-treatment liking		5.342 (0.407) ***	
Average pre-treatment sharing			5.562 (0.398) ***
Number of posts since treatment (centered)	-0.00738 (0.00135) ***	-0.00580 (0.00450)	-0.000424 (0.00673)
Condition : manipulative or not	-0.00675 (0.0313)	-0.141 (0.180)	-0.408 (0.199) *
Condition : avg. pre-treatment dwell time	-0.00730 (0.00473)		
Condition : avg. pre-treatment liking		-0.908 (0.468) .	
Condition : avg. pre-treatment sharing			-0.744 (0.654)
Condition : num. posts since treatment	-0.000676 (0.00193)	-0.00126 (0.00642)	-0.00313 (0.00998)
RMSE	1.090		
Adj. R2	0.176		
Log-likelihood		-2101.5	-1363.5
Adj. Pseudo R2		0.251	0.222
BIC		4274.4	2798.0
Squared Cor.		0.251	0.157

Table S26. Study 4: regression per outcome variable. Ad hominem manipulation technique. *p<0.05, **p<0.01, ***p<0.001

	(log) dwell time	like or not	share or not
(Intercept)	2.392 (0.0449) ***	-2.439 (0.236) ***	-3.0311 (0.149) ***
Condition (treatment: control)	-0.0549 (0.0576)	0.199 (0.103) .	-0.289 (0.233)
Manipulative or not (manipulative : neutral)	-0.0384 (0.0265)	-0.205 (0.254)	-0.306 (0.156) .
Average pre-treatment dwell time	0.0214 (0.00353) ***		
Average pre-treatment liking		5.378 (0.381) ***	
Average pre-treatment sharing			5.262 (0.394) ***
Number of posts since treatment (centered)	-0.006643 (0.00353) ***	-0.00959 (0.00434) *	-0.00495 (0.00592)
Condition : manipulative or not	0.0579 (0.0303) .	-0.185 (0.114)	-0.431 (0.265)
Condition : avg. pre-treatment dwell time	-0.00487 (0.00518)		
Condition : avg. pre-treatment liking		-0.798 (0.461) .	
Condition : avg. pre-treatment sharing			
Condition : num. posts since treatment	-0.00265 (0.00180)	0.00770 (0.00660)	-0.000503 (0.00797)
RMSE	1.0862		
Adj. R2	0.170		
Log-likelihood		-2082.6	-1281.1
Adj. Pseudo R2		0.259	0.253
BIC		4236.4	2633.3
Squared Cor.		0.254	0.158

Table S27. Study 2 attrition analysis: chi-square test comparing attrition of treatment and control groups. $\chi^2 = 0.112$, df = 1, p = 0.74, N = 993 were assigned a condition.

	Completed study	Did not complete study	Total
Treatment	486	22	508
Control	467	18	485
Total	953	42	993

Table S28. Study 2 attrition analysis: logistic regression predicting attrition (completed study or not) as a function of gender (reference level: male), age, income (0: less than $10,000, 11: $150,000 or more), ethnicity (reference level: White/Caucasian), area (0: rural, 2: urban), education level (0: less than high school degree, 6: doctoral or professional degree), belief in god (0: least belief to 7: most belief), feelings towards Republican voters (0: very cold, 100: very warm), feelings towards Democrat voters (0: very cold, 100: very warm), partisanship (0: strongly Republican, 6: strongly Democrat), political ideology (0: very Conservative, 6: very Liberal), supported 2024 US presidential candidate (reference level: Joe Biden), CRT (0: least cognitive reflection, 3: most cognitive reflection), AOT (1: least open-minded, 5: most open-minded), average dwell time per post pre-treatment, average number of likes pre-treatment, average number of shares pre-treatment. N = 973 completed Qualtrics, were assigned a condition, and got to the treatment video. We mark * as p<0.00054 for Bonferroni corrections.

	Estimate (SE)
Intercept	11.78 (12.043)
Gender (Female)	6.13 (4.95)
Gender (Other)	-49.47 (3151.29)
Age	-0.072 (0.068)
Ethnicity (Asian)	5.39 (5.21)
Ethnicity (Black or African American)	-1.026 (2.46)
Ethnicity (Other)	-2.68 (5.012)
Area	1.57 (1.71)
Income	0.17 (0.3)
Education	1.022 (0.83)
Belief in God	0.61 (0.35)
Affective Polarization (R)	0.02 (0.046)
Affective Polarization (D)	0.016 (0.05)
Partisanship	0.4 (0.83)
Political Ideology	0.52 (0.62)
Vote (Trump)	4.16 (3.44)
Vote (Not voting)	7.32 (5.69)
Vote (Prefer not to say)	4.65 (5.24)
Vote (Third party)	8.45 (23.56)
CRT	0.075 (0.87)
AOT	1.38 (1.1)
Average pre-treatment dwell time	2.81 (1.22)

Table S29. Study 3 attrition analysis: chi-square test comparing attrition of treatment and control groups. $\chi^2 = 0.053$, df = 1, p = 0.82, N = 987 were assigned a condition

	Completed study	Did not complete study	Total
Treatment	504	12	516
Control	458	13	471
Total	962	25	987

Table S30. Study 3 attrition analysis: logistic regression predicting attrition (completed study or not) as a function of gender (reference level: male), age, income (0: less than $10,000, 11: $150,000 or more), ethnicity (reference level: White/Caucasian), area (0: rural, 2: urban), education level (0: less than high school degree, 6: doctoral or professional degree), belief in god (0: least belief to 7: most belief), feelings towards Republican voters (0: very cold, 100: very warm), feelings towards Democrat voters (0: very cold, 100: very warm), partisanship (0: strongly Republican, 6: strongly Democrat), political ideology (0: very Conservative, 6: very Liberal), supported 2024 US presidential candidate (reference level: Joe Biden), CRT (0: least cognitive reflection, 3: most cognitive reflection), AOT (1: least open-minded, 5: most open-minded), average dwell time per post pre-treatment, average number of likes pre-treatment, average number of shares pre-treatment.. N = 973 completed Qualtrics, were assigned a condition, and got to the treatment video. We mark * as p<0.00054 for Bonferroni corrections.

	Estimate (SE)
Intercept	12.0 (6.36)
Gender (Female)	-0.53 (1.39)
Gender (Other)	11.39 (26150)
Age	0.012 (0.046)
Ethnicity (Asian)	-1.67 (1.99)
Ethnicity (Black or African American)	3.93 (2.22)
Ethnicity (Other)	17.55 (4134)
Area	0.17 (1.004)
Income	-0.016 (0.2)
Education	1.14 (0.6)
Belief in God	0.014 (0.027)
Affective Polarization (R)	-0.017 (0.038)
Affective Polarization (D)	0.0025 (0.034)
Partisanship	0.021 (0.79)
Political Ideology	-0.58 (0.62)
Vote (Trump)	-0.61 (2.13)
Vote (Not voting)	17.8 (2453)
Vote (Prefer not to say)	16.18 (6113)
Vote (Third party)	-2.83 (2.48)
CRT	-1.42 (0.69)
AOT	1.0 (0.85)
Average pre-treatment dwell time	0.91 (0.32)

Table S31. Study 4 attrition analysis: chi-square test comparing attrition of treatment and control groups. $\chi^2 = 0.023$, df = 1, p = 0.88, N = 967 were assigned a condition.

	Completed study	Did not complete study	Total
Treatment	474	14	488
Control	467	12	479
Total	941	26	967

Table S32. Study 4 attrition analysis: logistic regression predicting attrition (completed study or not) as a function of gender (reference level: male), age, income (0: less than $10,000, 11: $150,000 or more), ethnicity (reference level: White/Caucasian), area (0: rural, 2: urban), education level (0: less than high school degree, 6: doctoral or professional degree), belief in god (0: least belief to 7: most belief), feelings towards Republican voters (0: very cold, 100: very warm), feelings towards Democrat voters (0: very cold, 100: very warm), partisanship (0: strongly Republican, 6: strongly Democrat), political ideology (0: very Conservative, 6: very Liberal), supported 2024 US presidential candidate (reference level: Joe Biden), CRT (0: least cognitive reflection, 3: most cognitive reflection), AOT (1: least open-minded, 5: most open-minded), average dwell time per post pre-treatment, average number of likes pre-treatment, average number of shares pre-treatment. N = 961 completed Qualtrics, were assigned a condition, and got to the treatment video (used in logistic regression predicting attrition). We mark * as $p<0.00054$ for Bonferroni corrections.

	Estimate (SE)
Intercept	8.31 (2.27)*
Gender (Female)	-0.23 (0.52)
Gender (Other)	14.69 (4775)
Age	-0.014 (0.022)
Ethnicity (Asian)	0.029 (1.15)
Ethnicity (Black or African American)	0.26 (0.77)
Ethnicity (Other)	-0.51 (0.85)
Area	-0.6 (0.4)
Income	0.06 (0.084)
Education	-0.17 (0.21)
Belief in God	-0.087 (0.11)
Affective Polarization (R)	0.006 (0.012)
Affective Polarization (D)	-0.0019 (0.012)
Partisanship	-0.037 (0.26)
Political Ideology	0.032 (0.19)
Vote (Trump)	-1.025 (0.92)
Vote (Not voting)	1.02 (1.2)
Vote (Prefer not to say)	-0.56 (1.25)
Vote (Third party)	15.18 (1159)
CRT	-0.0006 (0.22)
AOT	-0.34 (0.3)
Average pre-treatment dwell time	0.11 (0.027)*

Table S33. Study 5 attrition analysis: chi-square test comparing attrition of treatment and control groups. $\chi^2 = 0.15$, df = 1, p = 0.69, N = 1016 were assigned a condition.

	Completed study	Did not complete study	Total
Treatment	514	13	527
Control	474	15	489
Total	988	28	1016

Table S34. Study 5 attrition analysis: logistic regression predicting attrition (completed study or not) as a function of gender (reference level: male), age, income (0: less than $10,000, 11: $150,000 or more), ethnicity (reference level: White/Caucasian) area (0: rural, 2: urban), education level (0: less than high school degree, 6: doctoral or professional degree), belief in god (0: least belief to 7: most belief), feelings towards Republican voters (0: very cold, 100: very warm), feelings towards Democrat voters (0: very cold, 100: very warm), partisanship (0: strongly Republican, 6: strongly Democrat), political ideology (0: very Conservative, 6: very Liberal), supported 2024 US presidential candidate (reference level: Joe Biden), CRT (0: least cognitive reflection, 3: most cognitive reflection), AOT (1: least open-minded, 5: most open-minded), average dwell time per post pre-treatment, average number of likes pre-treatment, average number of shares pre-treatment. N = 1012 completed Qualtrics, were assigned a condition, and got to the treatment video (used in logistic regression predicting attrition). We mark * as p<0.00054 for Bonferroni corrections.

	Estimate (SE)
Intercept	3.65 (2.11)
Gender (Female)	0.24 (0.51)
Gender (Other)	16.66 (2816)
Age	0.019 (0.022)
Ethnicity (Asian)	14.9 (897.5)
Ethnicity (Black or African American)	2.08 (1.1)
Ethnicity (Other)	-0.19 (0.81)
Area	-0.025 (0.35)
Income	0.15 (0.074)
Education	-0.099 (0.19)
Belief in God	0.015 (0.092)
Affective Polarization (R)	-0.013 (0.012)
Affective Polarization (D)	-0.00082 (0.011)
Partisanship	-0.26 (0.24)
Political Ideology	0.19 (0.18)
Vote (Trump)	-0.37 (0.84)
Vote (Not voting)	0.11 (0.78)
Vote (Prefer not to say)	-1.15 (0.91)
Vote (Third party)	0.75 (1.26)
CRT	-0.5 (0.2)
AOT	0.27 (0.28)
Average pre-treatment dwell time	0.067 (0.019)

Table S35. Study 4: regression per outcome variable. Including all techniques.

	(log) dwell time	like or not	share or not
(Intercept)	2.393 (0.039)***	-2.259 (0.116)***	-2.806 (0.117)***
Condition (treatment: control)	-0.07 (0.057)	0.173 (0.089)	-0.06 (0.119)
Manipulative or not (manipulative : neutral)	-0.014 (0.007)	-0.245 (0.124)*	-0.085 (0.13)
Average pre-treatment dwell time	0.023 (0.003)***		
Average pre-treatment liking		5.121 (0.256)***	
Average pre-treatment sharing			5.057 (0.292)***
Number of posts since treatment (centered)	-0.007 (0.0007)***	-0.009 (0.002)***	-0.008 (0.002)***
Condition : manipulative or not	0.012 (0.009)	-0.194 (0.074)**	-0.187 (0.084)*
Condition : avg. pre-treatment dwell time	-0.007 (0.005)		
Condition : avg. pre-treatment liking		-0.645 (0.35)	
Condition : avg. pre-treatment sharing			0.035 (0.484)
Condition : num. posts since treatment	-0.001 (0.001)	0.0003 (0.003)	0.003 (0.003)
RMSE	1.097		
Adj. R2	0.168		
Log-likelihood		-14,175.9	-10,467
Adj. Pseudo R2		0.242936	0.215

BIC	28,437.9	21,020.1
Squared Cor.	0.243142	0.164

Table S36. Study 4: OLS regression predicting (log) dwell time and logistic regressions predicting like or not and share or not as a function of the inoculation treatment for posts containing any manipulation technique with robust standard errors clustered on participants and posts. *p<0.05, **p<0.01, ***p<0.001

	(log) dwell time	like or not	share or not
(Intercept)	2.378 (0.039)***	-2.544 (0.129)***	-2.907 (0.146)***
Condition (treatment: control)	-0.058 (0.057)	-0.014 (0.111)	-0.263 (0.135) *(p = 0.051)*
Average pre-treatment dwell time	0.023 (0.003)***		
Average pre-treatment liking		5.315 (0.312)***	
Average pre-treatment sharing			5.132 (0.32)***
Number of posts since treatment (centered)	-0.007 (0.0008)***	-0.009 (0.002)***	-0.01 (0.003)***
Condition : avg. pre-treatment dwell time	-0.008 (0.005)		
Condition : avg. pre-treatment liking		-0.658 (0.414)	
Condition : avg. pre-treatment sharing			0.118 (0.531)
Condition : num. posts since treatment	-0.00006 (0.0013)	-0.0005 (0.004)	0.007 (0.005)
RMSE	1.093		
Adj. R2	0.169		
Log-likelihood		-6,431.2	-4,950.5
Adj. Pseudo R2		0.26	0.227

BIC		12,922.9	9,961.4
Squared Cor.		0.257	0.167

Table S37. Study 4: OLS regression predicting (log) dwell time and logistic regressions predicting like or not and share or not as a function of the inoculation treatment for posts containing any non-manipulation technique with robust standard errors clustered on participants and posts. *p<0.05, **p<0.01, ***p<0.001

	(log) dwell time	like or not	share or not
(Intercept)	2.393 (0.039)***	-2.229 (0.124)***	-2.791 (0.123)***
Condition (treatment: control)	-0.07 (0.057)	0.165 (0.089)	-0.051 (0.117)
Average pre-treatment dwell time	0.022 (0.003)***		
Average pre-treatment liking		4.947 (0.277)***	
Average pre-treatment sharing			4.989 (0.321)***
Number of posts since treatment (centered)	-0.007 (0.0008)***	-0.009 (0.002)***	-0.006 (0.003)*
Condition : avg. pre-treatment dwell time	-0.006 (0.005)		
Condition : avg. pre-treatment liking		-0.627 (0.34)	
Condition : avg. pre-treatment sharing			-0.042 (0.484)
Condition : num. posts since treatment	-0.002 (0.001)	0.0009 (0.003)	-0.001 (0.005)
RMSE	1.099		
Adj. R2	0.167		
Log-likelihood		-7,739.9	-5,514
Adj. Pseudo R2		0.224	0.203
BIC		15,540.3	11,088.4
Squared Cor.		0.23	0.161

Table S38. Primary analysis for Study 4: OLS regression predicting (log) dwell time and logistic regressions predicting like or not and share or not as a function of the inoculation treatment for posts containing **emotional manipulation only** with robust standard errors clustered on participants and posts. *p<0.05, **p<0.01, ***p<0.001

	(log) dwell time	like or not	share or not
(Intercept)	2.398 (0.042)***	-2.594 (0.223)***	-2.465 (0.191)***
Condition (treatment: control)	-0.047 (0.058)	-0.028 (0.145)	-0.202 (0.144)
Average pre-treatment dwell time	0.022 (0.003)***		
Average pre-treatment liking		5.25 (0.44)***	
Average pre-treatment sharing			5.141 (0.408)***
Number of posts since treatment (centered)	-0.006 (0.001)***	-0.015 (0.004)**	
Condition : avg. pre-treatment dwell time	-0.007 (0.005)		
Condition : avg. pre-treatment liking		-0.586 (0.54)	
Condition : avg. pre-treatment sharing			-0.010 (0.003)**
Condition : num. posts since treatment	-0.0002 (0.002)	0.003 (0.007)	0.0004 (0.006)
RMSE	1.086		
Adj. R2	0.158		
Log-likelihood		-2,295.2	-2,302.1
Adj. Pseudo R2		0.26	0.213
BIC		4,644.7	4,658.5
Squared Cor.		0.254	0.188

Table S39. Primary analysis for Study 4: OLS regression predicting (log) dwell time and logistic regressions predicting like or not and share or not as a function of the inoculation treatment for posts containing emotionally **non-manipulative posts only** with robust standard errors clustered on participants and posts. *p<0.05, **p<0.01, ***p<0.001

	(log) dwell time	like or not	share or not
(Intercept)	2.398 (0.041)***	-2.453 (0.267)***	-2.498 (0.194)***
Condition (treatment: control)	-0.049 (0.058)	0.162 (0.129)	-0.105 (0.15)
Average pre-treatment dwell time	0.022 (0.003)***		
Average pre-treatment liking		5.103 (0.46)***	
Average pre-treatment sharing			4.878 (0.384)***
Number of posts since treatment (centered)	-0.005 (0.001)***	-0.007 (0.005)	-0.002 (0.002)
Condition : avg. pre-treatment dwell time	-0.005 (0.005)		
Condition : avg. pre-treatment liking		-0.605 (0.357)	
Condition : avg. pre-treatment sharing			-0.103 (0.497)
Condition : num. posts since treatment	-0.003 (0.001)*	0.003 (0.006)	-0.004 (0.005)
RMSE	1.094		
Adj. R2	0.165		
Log-likelihood		-2,547.0	-2,309.7
Adj. Pseudo R2		0.239	0.19
BIC		5,148.2	4,673.7
Squared Cor.		0.241	0.166

Table S40. Primary analysis for Study 5: OLS regression predicting (log) dwell time and logistic regressions predicting like or not and share or not as a function of the inoculation treatment for posts containing **emotional manipulation only** with robust standard errors clustered on participants and posts. *p<0.05, **p<0.01, ***p<0.001

	(log) dwell time	like or not	share or not
(Intercept)	2.466 (0.041)***	-2.459 (0.188)***	-2.271 (0.181)***
Condition (treatment: control)	0.005 (0.059)	0.005 (0.102)	-0.155 (0.133)
Average pre-treatment dwell time	0.005 (0.005)		
Average pre-treatment liking		4.124 (0.322)***	
Average pre-treatment sharing			4.849 (0.371)***
Number of posts since treatment (centered)	-0.013 (0.003)***	-0.014 (0.01)	-0.001 (0.005)
Condition : avg. pre-treatment dwell time	0.019 (0.008)*		
Condition : avg. pre-treatment liking		1.112 (0.333)***	
Condition : avg. pre-treatment sharing			0.08 (0.483)
Condition : num. posts since treatment	-0.002 (0.005)	-0.002 (0.014)	-0.0008 (0.009)
RMSE	1.055		
Adj. R2	0.13		
Log-likelihood		-2,934.6	-2,993.1
Adj. Pseudo R2		0.22	0.202
BIC		5,924.4	6,041.4
Squared Cor.		0.214	0.189

Table S41. Primary analysis for Study 5: OLS regression predicting (log) dwell time and logistic regressions predicting like or not and share or not as a function of the inoculation treatment for posts containing emotionally **non-manipulative posts only** with robust standard errors clustered on participants and posts. *p<0.05, **p<0.01, ***p<0.001

	(log) dwell time	like or not	share or not
(Intercept)	2.481 (0.042)***	-2.328 (0.268)***	-2.542 (0.178)***
Condition (treatment: control)	0.006 (0.061)	0.074 (0.107)	0.037 (0.114)
Average pre-treatment dwell time	0.006 (0.005)		
Average pre-treatment liking		4.429 (0.449)***	
Average pre-treatment sharing			4.879 (0.419)***
Number of posts since treatment (centered)	-0.015 (0.003)***	-0.005 (0.008)	0.017 (0.009)
Condition : avg. pre-treatment dwell time	0.019 (0.008)*		
Condition : avg. pre-treatment liking		0.508 (0.363)	
Condition : avg. pre-treatment sharing			0.225 (0.455)
Condition : num. posts since treatment	0.001 (0.003)	-0.009 (0.013)	-0.26 (0.011)*
RMSE	1.05		
Adj. R2	0.134		
Log-likelihood		-3,158.2	-2,760.6
Adj. Pseudo R2		0.219	0.212
BIC		6,371.6	5,576.4
Squared Cor.		0.217	0.189
